# A cortico-hypothalamic circuit regulates metabolism and stress resilience in a sex-specific manner

**DOI:** 10.1016/j.molmet.2026.102459

**Published:** 2026-10-22

**Authors:** Vasilios Drakopoulos, Xiaolong Feng, Daria J. Paulis, Abigail C. Wust, Lotus S. Jeffs, Romana Stark, Greg J. Stuart, Claire J. Foldi, Alex Reichenbach, Zane B. Andrews

**Affiliations:** Monash Biomedicine Discovery Institute and Department of Physiology, Monash University, Clayton, Victoria, Australia

**Keywords:** Cortex, Hypothalamus, Bodyweight, Stress, Hedonia, Behaviour

## Abstract

**Objective:**

The neural mechanisms controlling food intake and body weight have traditionally been ascribed to neuroendocrine feedback acting within the hypothalamus. However, recent studies highlight that integration of external sensory stimuli within cortical regions also regulates energy metabolism, raising the possibility that sensory input can override homeostatic control of feeding behaviour.

**Methods:**

In this study, we examined whether a pathway from the medial prefrontal cortex (mPFC) to the lateral hypothalamus (LH), which responds to acute stressors and suppresses food intake, also produces long-term effects on metabolism and stress-induced behaviour. Manipulation of this pathway was achieved through caspase-mediated chronic ablation or NaChBac-mediated chronic activation in mice.

**Results:**

Chronic mPFC-LH ablation increased body weight gain and lean mass in chow-fed males, but not females, and improved glucose clearance in males and insulin sensitivity in females. In contrast, chronic mPFC-LH activation with NaChBac reduced body weight gain and improved glucose handling specifically in high fat diet (HFD)-fed females. Behaviourally, mPFC-LH ablation reduced exploration following acute stress, increased freezing during stress, and decreased hedonic processing in chow-fed males. Conversely, chronic mPFC-LH activation increased exploration after stress and lowered plasma corticosterone levels in males, and increased hedonic responses in chow-fed females, suggesting enhanced behavioural resilience.

**Conclusion:**

Collectively, these findings demonstrate that chronic manipulation of the mPFC-LH pathway produces reciprocal, sex- and diet-specific effects on body weight and glucose metabolism, as well as regulating behavioural resilience to acute stress. This novel top-down, cortico-hypothalamic pathway may play an important role in the sex-specific pathogenesis of metabolic and stress-related neuropsychiatric disorders.

## Highlights

• mPFC-LH manipulation drives diet- and sex-specific metabolic and behavioural effects.
• mPFC-LH ablation increases bodyweight gain in chow fed males.
• mPFC-LH activation decreases bodyweight gain in high fat diet fed females.
• mPFC-LH ablation reduces exploration after stress and increases freezing in males.
• mPFC-LH activation increases exploration after stress and hedonia in females.

## 1 Introduction

Obesity is the abnormal accumulation of fat caused by increased energy intake relative to expenditure [1]. It is a major worldwide problem that leads to many diseases such as diabetes and cancer [2]. With global levels rising, understanding the biology of body weight regulation is essential to combat this problem. Genome-wide association studies (GWAS) reveal that many genetic variants associated with obesity are found in the central nervous system, highlighting the importance of the brain for the control of energy homeostasis [3], [4], [5]. Many of the genes identified by GWAS are found in the hypothalamus and brainstem [6], [7] and known for their essential roles to integrate neuroendocrine and gut-brain feedback to maintain energy homeostasis [8]. However, in humans, excessive food consumption is also linked to environmental and cognitive cues predicting caloric availability and palatability [9]. In support of this, a combined single-cell RNA seq/GWAS approach identified numerous neuronal populations in the cortex, midbrain, thalamus and hippocampus, in addition to the hypothalamus and brainstem, that were significantly enriched for body mass index heritability [10]. This suggests other brain regions controlling the integration of sensory stimuli, learning, and memory act together with homeostatic systems to influence appetite and body weight [10].

As the cortex is essential for integrating sensory information, it may play an important role in linking sensory cues associated with food to hypothalamic regions controlling energy homeostasis. Indeed, inflammatory pain suppresses feeding through an excitatory circuit from the anterior cingulate cortex (ACC) to glutamatergic neurons in the lateral hypothalamus (LH), which activate satiety-signalling POMC neurons (13). Moreover, anterior insular cortex excitatory neurons respond to aversive visceral stimuli and suppress food intake by activating LH^Vglut2^ neurons [11]. These studies identify the LH as a key neural link between the cortex and the hypothalamus, an idea supported by electrophysiological studies showing that the majority of synaptic input to the LH originates from outside the LH [12], [13]. Moreover, LH glutamate and GABA neurons play essential roles in food intake and body weight, since the ablation of LH^Vglut2^ neurons results in elevated food intake and body weight gain, whereas ablation of LH^Vgat^ neurons reduces food intake and body weight [14], [15], [16].

Consistent with the role of the LH as a gateway for sensory inputs to the hypothalamus, we recently used spectral dynamic causal modelling of functionally connected human brain regions to identify a novel mPFC-LH pathway associated with BMI and metabolic state [17]. *In vivo* population calcium imaging found that mPFC-LH neurons respond to acute stressors with chemogenetic activation suppressing food intake. In contrast, chemogenetic inhibition of mPFC-LH neurons acutely increased novel and familiar food intake and motivated food seeking in a novel environment. Furthermore, mPFC projections to the LH are activated in cue-associated feeding paradigms [18]. Collectively, these studies highlight a novel pathway from the mPFC to the LH that responds to sensory cues and controls acute food intake and feeding behaviour. However, the impact of the mPFC-LH pathway on long-term body weight, body composition, glucose homeostasis, and stress related behaviour has not been explored. This pathway may play an important role in obesity as lesion or ablation of LH neurons affects long term body weight [19], [20]. Given the established sex differences in hypothalamic feeding circuits and metabolic responses to dietary challenge [21], [22], [23], we utilized two chronic approaches to modulate mPFC-LH function. Chronic caspase-mediated mPFC-LH ablation and chronic NaChBac-mediated activation were used to investigate the mPFC-LH pathways control on energy homeostasis and stress-related behaviour.

## 2 Results

### 2.1 Ablation of the mPFC-LH pathway causes sex-specific weight gain and blood glucose increase in chow fed males only

To target the mPFC-LH pathway, we used a dual viral approach with retro AAV-Cre injected into the LH and cre-dependent AAV-Caspase injected into the mPFC in both hemispheres (Figure 1A). We used this approach in fs-TRAP mice, which causes GFP expression in a cre-dependent manner, to validate caspase-mediated deletion of the mPFC-LH pathway. Caspase significantly reduced the number of GFP-positive cells in the mPFC-LH pathway compared to controls (Figure 1B,C). To assess the impact of this pathway on body weight and glucose homeostasis, we used cre-dependent caspase to ablate mPFC-LH neurons in C57BL/6 wild-type mice and then monitored body weight and composition along with glucose and insulin tolerance tests (GTT, ITT) over approximately 20 weeks (Figure 1D,E). There was a significant increase in bodyweight gain over time in mPFC-LH^caspase^ males on a chow diet compared to controls (two-way ANOVA main effect of days (F (2.279, 29.63) = 55.43, p < 0.0001), group (F (1, 13) = 7.432, p = 0.0173) and interaction (F (2.279, 29.63) = 3.320, p = 0.0443; Figure 1F, Supp. Fig. 1A). In contrast, no bodyweight gain over time was observed in mPFC-LH^caspase^ females on a chow diet compared to controls (Figure 1G, Supp. 1 B). Although no significant differences in fat mass were detected in mPFC-LH^caspase^ males on a chow diet compared to controls (Fig 1H), there was a significant increase in lean mass gain at weeks 8 and 16, compared to controls (Figure 1J, Supp 1 E). Despite no significant differences in overall weight gain, mPFC-LH^caspase^ females on a chow diet had a significant increase in lean mass gain at weeks 8 and 16 with no difference in fat mass (Figure 1I,K). We found no significant differences in food intake as measured by BioDaq feeding cages in either males or females between groups (Supp. Fig. 2). However, these experiments were conducted approximately 24–28 weeks after surgery when body weight had already diverged.

**Figure 1:**
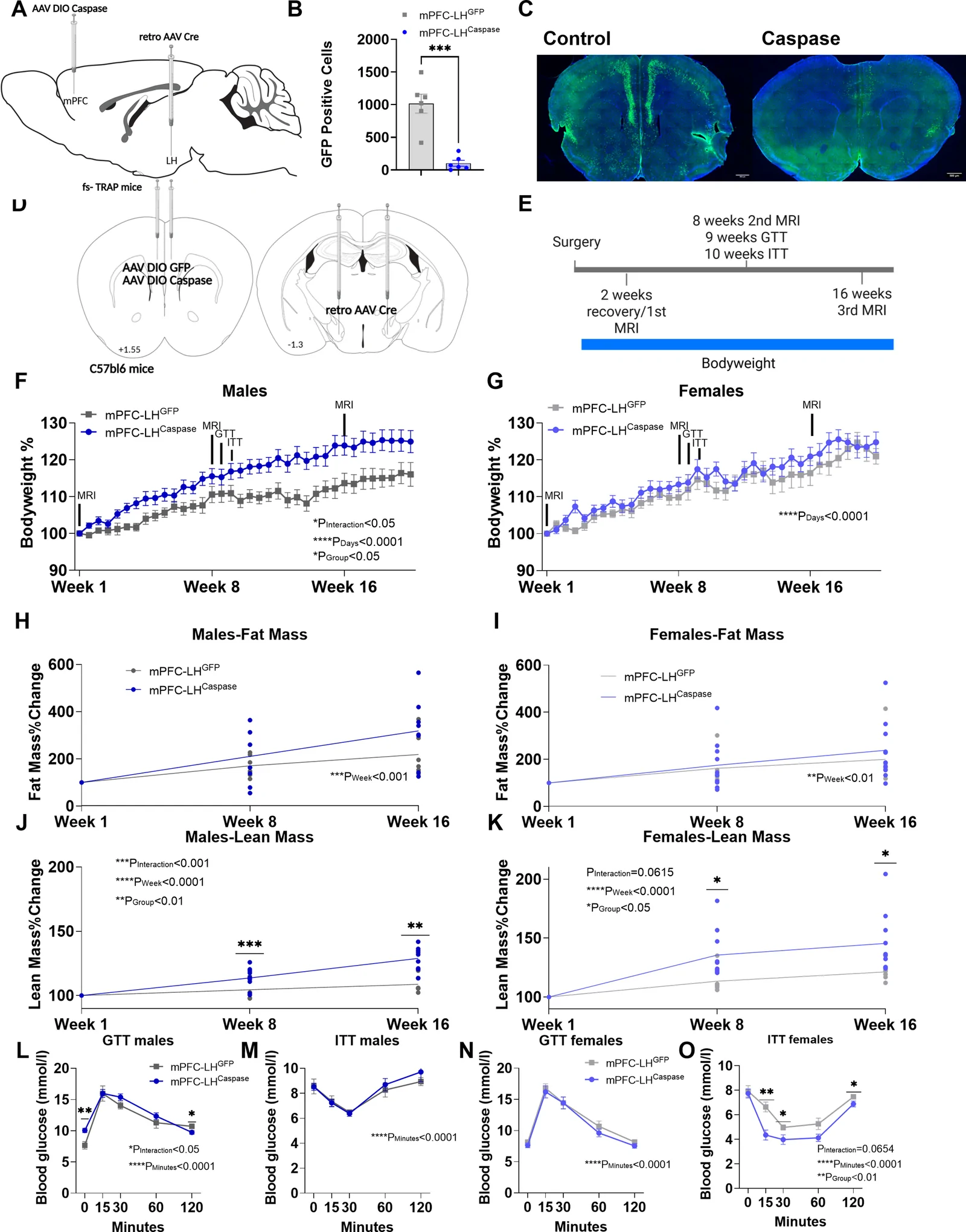
**mPFC-LH caspase males on chow have higher bodyweight gain and both sexes have more lean mass gain. (A)** Schematic of a sagittal section showing viral injections in fs- TRAP mice for caspase validation. **(B)** Number of GFP positive cells for validation of caspase. **(C)** Microscope images showing GFP expression in control and caspase fs- TRAP mice for validation. **(D)** Two coronal sections showing viral injections in the LH and mPFC. **(E)** Schematic of a timeline for surgery and subsequent bodyweight measurements, MRIs, GTT, and ITT. **(F)** Percent bodyweight gain of control and caspase males. **(G)** Percent bodyweight gain of control and caspase females. **(H)** Percent fat mass gain for control and caspase males. **(I)** Percent fat mass change gain for control and caspase females. **(J)** Percent lean mass gain for control and caspase males. **(K)** Percent lean mass gain for control and caspase females. **(L)** Glucose tolerance test (GTT) of control and caspase males. **(M)** Insulin tolerance test (ITT) of control and caspase males. **(N)** GTT of control and caspase females. **(O)** ITT of control and caspase females. Welch's t-test **(B)** with *n* = 6 for both controls and caspase. Two-way repeated-measures ANOVA with Šídák's multiple comparisons test **(F, G, H, I, J, K, L, M, N, O)**. For males **(F, J, L, M**) *n* = 9 for caspase, **(H)***n* = 8 for caspase, and *n* = 6 for all controls. For females **(G, I, K)***n* = 10 for caspase, **(N, O)***n* = 9 for caspase, and *n* = 6 for all controls. Data is represented as mean ± standard error of the mean (SEM), ⁎p < 0.05, ⁎⁎p < 0.01, ⁎⁎⁎p < 0.001.

Fasting blood glucose was significantly higher in mPFC-LH^caspase^ males on a chow diet (GTT time 0) compared to controls but was significantly lower 120 min following glucose (two-way ANOVA main effect of minutes (F (2.533, 32.93) = 82.54, p < 0.0001) and a significant minute × group interaction (F (2.533, 32.93) = 3.811, p = 0.0241); Fig 1L). Despite higher starting blood glucose at time 0, area under the curve (AUC) was significantly lower in mPFC-LH^caspase^ males on a chow diet (Supp. Fig. 1G), suggesting mPFC-LH^caspase^ males had better glucose clearance. There were no differences during an ITT between groups in males (Figure 1M, Supp. Fig. 1H). No significant difference in glucose clearance during a GTT was observed in mPFC-LH^caspase^ females on a chow diet compared to controls (Fig 1N). However, during an ITT mPFC-LH^caspase^ females had significantly lower blood glucose compared to controls at timepoints 15, 30, and 120 min after insulin treatment (two-way ANOVA, main effects for minutes and group and a significant interaction; Fig 1O), suggesting greater insulin sensitivity.

We then explored if this weight gain ensues in HFD fed mice. There were no significant differences in bodyweight gain in either mPFC-LH^caspase^ males or females compared to controls fed on a high-fat diet (HFD) (Figure 2A,B). mPFC-LH^caspase^ males on HFDs had a non-significant trend towards lower fat mass with no effect on lean mass change (Figure 2C,E). mPFC-LH^caspase^ females fed on HFD had no significant differences in fat or lean mass change (Figure 2D,F). There were no significant differences in glucose handling or insulin sensitivity during a GTT and ITT between mPFC-LH^caspase^ males or females compared to controls fed on HFD, although there is a non-significant trend towards a lower ITT area above the curve (AAC) in males (Figure 2G,H; Supp. Fig. 1R) and a non-significant interaction trend (two-way ANOVA; F (3.009, 39.12) = 2.392, p = 0.083) in females during an ITT (Figure 2I,J). The absence of weight and glucose effects under HFD suggests that the metabolic contribution of the mPFC-LH pathway is engaged specifically under conditions of low dietary stress, pointing to a state-dependent role for cortical control of hypothalamic feeding circuits.

**Figure 2:**
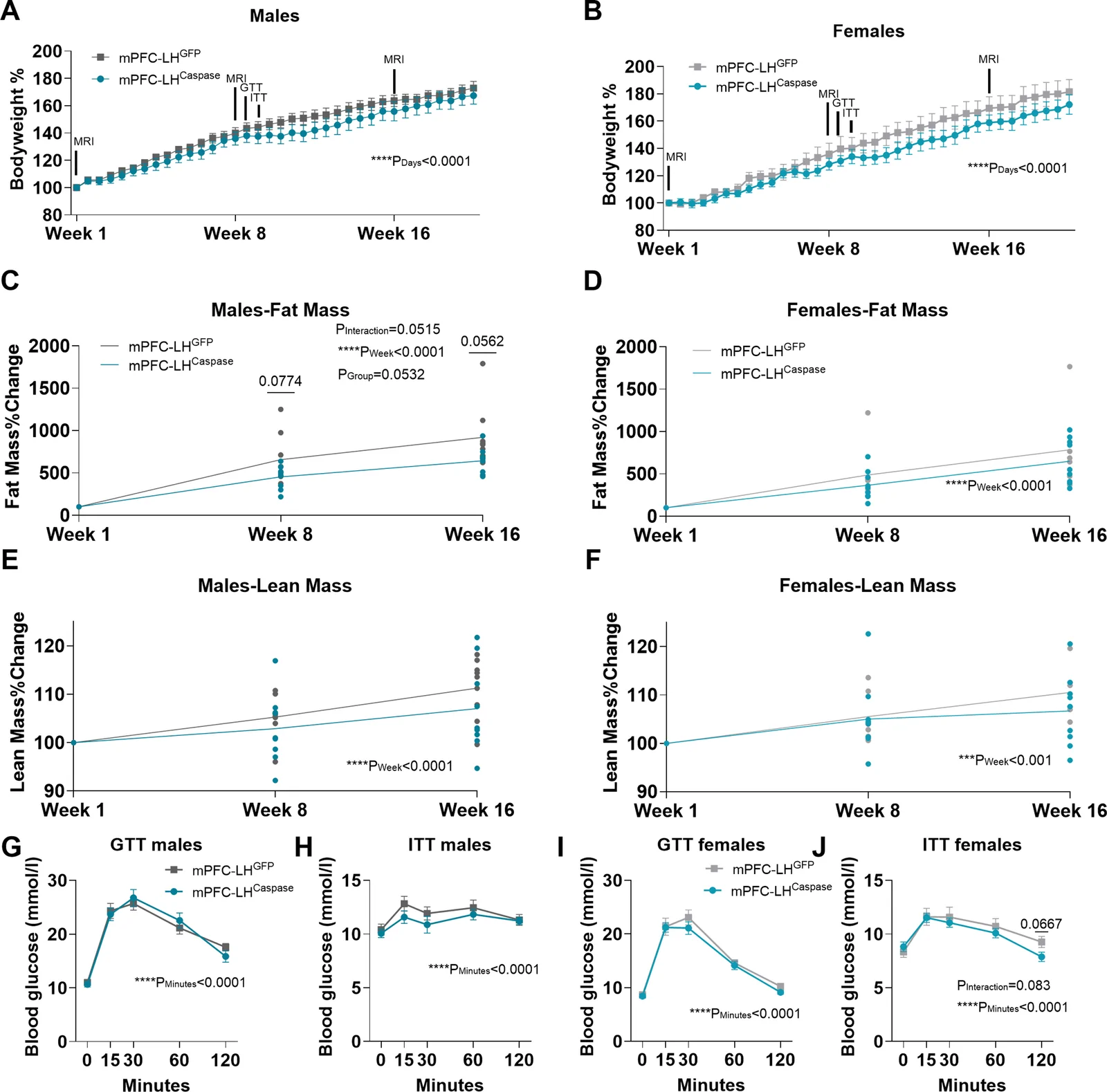
**mPFC-LH caspase mice on HFD have no metabolic differences. (A)** Percent bodyweight gain of control and caspase males. **(B)** Percent body weight gain of control and caspase females. **(C)** Percent fat mass gain for control and caspase males. **(D)** Percent fat mass gain for control and caspase females. **(E)** Percent lean mass gain for control and caspase males. **(F)** Percent lean mass gain for control and caspase females. **(G)** Glucose tolerance test (GTT) of control and caspase males. **(H)** Insulin tolerance test (ITT) of control and caspase males. **(I)** GTT of control and caspase females. **(J)** ITT of control and caspase females. Two-way repeated-measures ANOVA with Šídák's multiple comparisons test **(A, B, C, D, E, F, H, I, J)** and mixed-effects analysis with Šídák's multiple comparisons test **(G).** For males **(A)***n* = 8 for caspase, **(C, E, G, H)***n = 9* for caspase, and *n = 9* for all controls. For females **(B, D, F, I, J)***n* = 9 for caspase and *n* = 6 for controls. Data is represented as mean ± SEM.

### 2.2 Activation of the mPFC-LH pathway attenuates HFD-associated weight gain in female but not male mice

The data above indicate that mPFC-LH pathway ablation has sex- and diet-specific effects on body weight, body composition and glucose homeostasis. We next determined if chronic activation of the mPFC-LH produced opposing responses. Again, we used a dual viral approach with retro AAV-Cre injected into the LH and cre-dependent AAV NaChBac or AAV mCherry injected into the mPFC (Figure 3A–C). NaChBac is a bacterial sodium channel that increases firing and neural activity within transduced neurons [24], which has previously been used to chronically activate hypothalamic feeding circuits and affect body weight and body composition [25].

**Figure 3:**
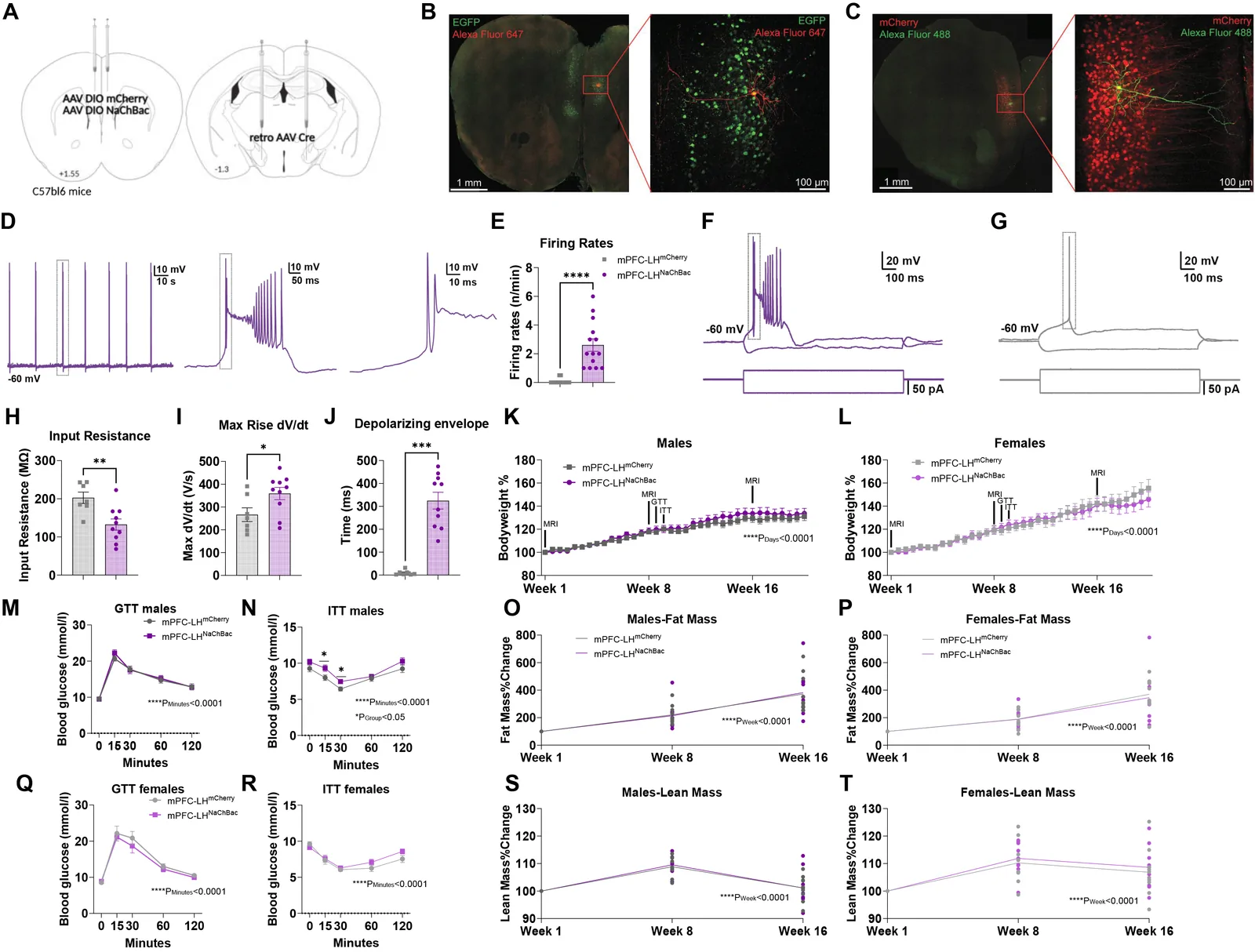
**mPFC-LH NaChBac mice on chow show no differences in metabolic function. (A)** Two coronal sections showing viral injections in the LH and mPFC. **(B)** Representative images showing EGFP expression in the mPFC from a NaChBac mouse (Green). A biocytin-filled recorded neuron is shown (Red). **(C)** Representative images showing mCherry expression in the mPFC of a control mouse (Red). A biocytin-filled recorded neuron is shown (Green). **(D)** Representative whole-cell current-clamp recording showing spontaneous firing in a NaChBac-expressing mPFC-LH neuron at the resting membrane potential. **(E)** Quantification of the spontaneous firing in control and mPFC-LH^NaChBac^ neurons. **(F,G)** Representative whole-cell current-clamp recording from mPFC-LH^NaChBac^ (F) and control (G) mPFC-LH neurons during a 1 s hyperpolarising and depolarising current steps. **(H)** Input resistance of control and mPFC-LH^NaChBac^ neurons. **(I)** Action potential maximum dV/dt of control and mPFC-LH^NaChBac^ neurons. **(J)** Depolarization duration of control and mPFC-LH^NaChBac^ neurons. **(K)** Percent bodyweight gain of control and NaChBac males. **(L)** Percent bodyweight gain of control and NaChBac females. **(M)** Glucose tolerance test (GTT) of control and NaChBac males. **(N)** Insulin tolerance test (ITT) of control and NaChBac males. **(O)** Percent fat mass gain for control and NaChBac males. **(P)** Percent fat mass gain for control and NaChBac females. **(Q)** GTT of control and NaChBac females. **(R)** ITT of control and NaChBac females. **(S)** Percent lean mass gain for control and NaChBac males. **(T)** Percent lean mass gain for control and NaChBac females. Mann–Whitney test **(D, H)**, unpaired t test **(I, J),** Two-way repeated-measures ANOVA with Šídák's multiple comparisons test **(K, L, M, N, O, P, Q, R, S, T)**. For controls and NaChBac each **(E)***n* = 14. For controls **(H, I, J)***n* = 7 and NaChBac *n* = 10. For males **(K, M, N, O, S)***n* = 10 for controls, and *n* = 9 for NaChBac. For females **(L, P, Q, R, T)***n* = 10 for controls, and *n* = 9 for NaChBac. All electrophysiological data is from individual cells from *n* = 1 from each group where mice were 10 weeks of age conducted 2 weeks after surgery. Data is represented as mean ± SEM, ⁎p < 0.05, ⁎⁎p < 0.01, ⁎⁎⁎p < 0.001, ⁎⁎⁎⁎p < 0.0001.

Histology confirmed EGFP-labelled mPFC-LH^NaChBac^ or mPFC-LH^mCherry^ control neurons in the mPFC and enabled targeted whole-cell current-clamp recordings from mPFC-LH projecting neurons in brain slices (Figure 3B,C). mPFC-LH^NaChBac^ neurons fired spontaneously at resting membrane potentials and displayed prolonged depolarizations with burst-like firing (Fig 3D), whereas control mPFC-LH^mCherry^ neurons showed little spontaneous activity (Fig 3E). Just suprathreshold depolarizing current steps evoked a similar firing pattern in mPFC-LH^NaChBac^ neurons, with prolonged depolarization and multiple spikes, whereas control mPFC-LH^mCherry^ neurons typically produced a single action potential (Figure 3F,G). Investigation of intrinsic electrophysiological properties during somatic current injection indicated that mPFC-LH^NaChBac^ neurons had a significantly lower input resistance, faster rising action potentials and longer depolarizing envelope during action potential firing compared to control mPFC-LH^mCherry^ neurons (Figure 3H–J; Supp. Fig. 3A and B), consistent with increased intrinsic excitability in mPFC-LH^NaChBac^ neurons. No differences in resting membrane potential, rheobase, action potential threshold, action potential amplitude or action potential half-width were found between mPFC-LH^NaChBac^ and mPFC-LH^mCherry^ neurons (Supp. Fig. 3C–G).

Given the sex-dependent differences in the impact of caspase ablation of the mPFC-LH pathway described above, we compared the intrinsic electrophysiological properties of mPFC-LH^NaChBac^ neurons between males and females. Representative recordings from female and male mPFC-LH^NaChBac^ neurons showed similar firing patterns, with spontaneous firing at resting membrane potential with a prolonged depolarizing voltage envelope during current injection (Figure 3B,D, F; Supp. Fig. 4A–C). Average action potential waveforms aligned to the peak were similar between males and females (Supp. Fig. 4D). Consistent with this, there were no significant differences in the resting membrane potential, input resistance or spontaneous action potential firing rate between sexes (Supp. Fig. 4E–M). There were also no significant differences in action potential rate of rise (dV/dt), amplitude or half-width, or the depolarizing voltage envelope duration during suprathreshold current injection between sexes (Supp. Fig. 4E–M), although these studies may have been under powered to detect sex differences.

There were no significant differences in body weight of mPFC-LH^NaChBac^ mice compared to controls on a chow diet regardless of sex (Figure 3K, L, Supp. Fig. 5A and B). Similarly, mPFC-LH^NaChBac^ expression did not affect glucose clearance in either male or female mice during a GTT compared to mPFC-LH^mCherry^ mice (Figure 3M,Q). Activation of the mPFC-LH pathway with NaChBac transiently reduced insulin sensitivity during an ITT, as assessed by higher blood glucose at 15 and 30 min after insulin injection in male mice compared to controls (two-way ANOVA main effect of group F (1, 17) = 4.918, p = 0.0405, Fig 3N). There were no differences in mPFC-LH^NaChBac^ and mPFC-LH^mCherry^ female mice during an ITT (Fig 3R). We also observed no differences in fat mass or lean mass gain in mPFC-LH^NaChBac^ or mPFC-LH^mCherry^ male and female mice compared to controls (Figure 3O, P, S, T). Collectively, these studies show that chronic activation of the mPFC-LH pathway has no effect on body weight and composition in males or females, but acutely reduces insulin sensitivity in males.

We next tested the impact of activation of the mPFC-LH in mice fed HFD. In response to HFD, mPFC-LH^NaChBac^ or mPFC-LH^mCherry^ male mice gained weight at the same rate (Figure 4A), however diet-induced weight gain was attenuated in female mPFC-LH^NaChBac^ mice compared to mPFC-LH^mCherry^ controls (two-way ANOVA, interaction of time and group F (35, 630) = 2.568, p < 0.0001; Figure 4A,B). There were non-significant trends towards lower fat mass in mPFC-LH^NaChBac^ male mice with no differences in lean mass change (Figure 4C,E). mPFC-LH^NaChBac^ females on HFD had no significant differences in fat or lean mass change compared to mPFC-LH^mCherry^ controls (Figure 4D,F). No difference between HFD-fed mPFC-LH^NaChBac^ and mPFC-LH^mCherry^ male mice was observed during a GTT and ITT, (Figure 4G,H), however, HFD-fed mPFC-LH^NaChBac^ females had significantly lower fasting blood glucose during a GTT and ITT, and lower blood glucose at the 15-minute timepoint after glucose injection (Figure 4I,J).

**Figure 4:**
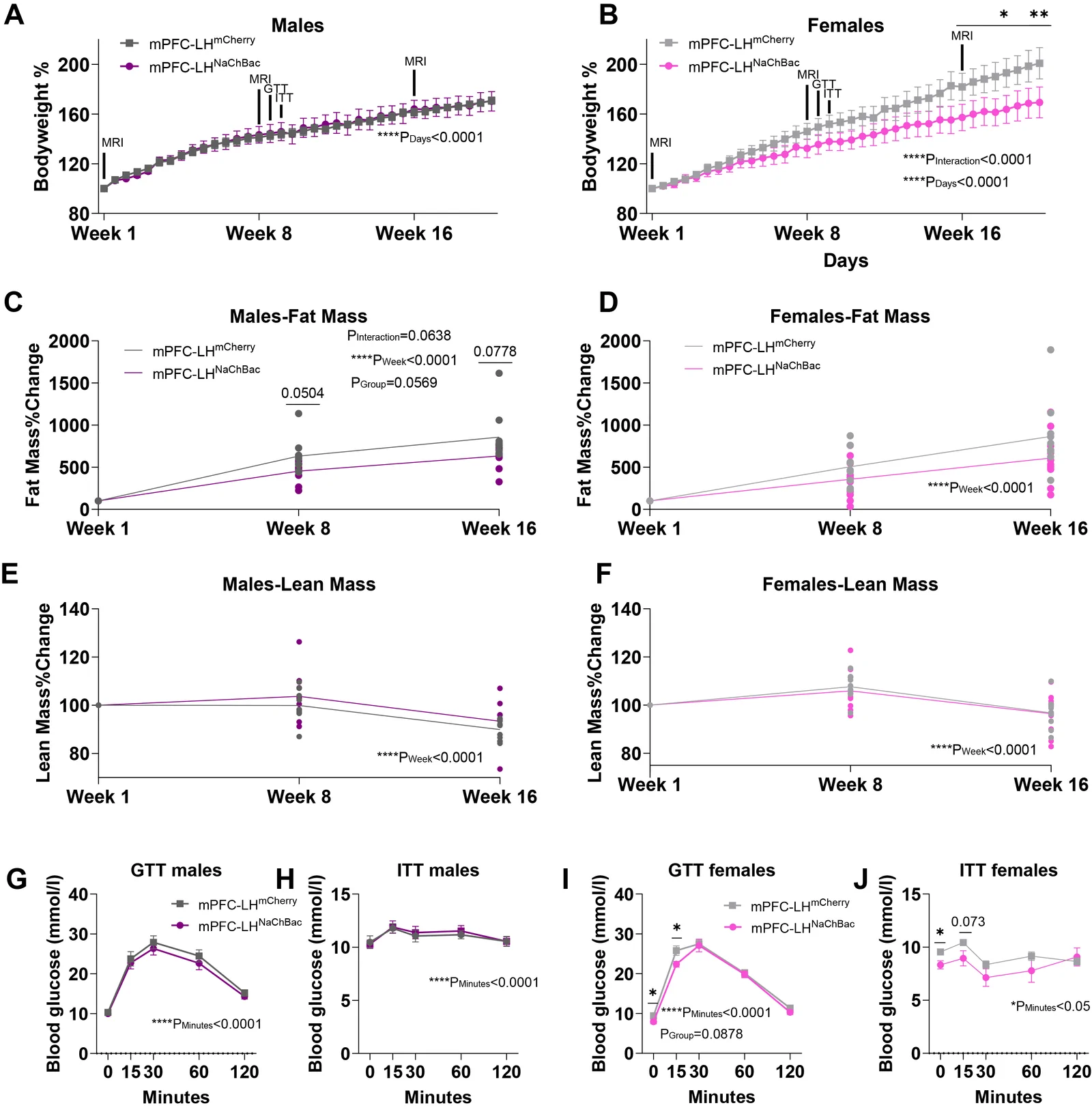
**mPFC-LH NaChBac females on HFD have a lower bodyweight gain over time and fasting blood glucose. (A)** Percent bodyweight gain of control and NaChBac males. **(B)** Percent bodyweight gain of control and NaChBac females. **(C)** Percent fat mass gain for control and NaChBac males. **(D)** Percent fat mass gain for control and NaChBac females. **(E)** Percent lean mass gain for control and NaChBac males. **(F)** Percent lean mass gain for control and NaChBac females. **(G)** Glucose tolerance test (GTT) of control and NaChBac males. **(H)** Insulin tolerance test (ITT) of control and NaChBac males. **(I)** GTT of control and NaChBac females. **(J)** ITT of control and NaChBac females. Two-way repeated-measures ANOVA with Šídák's multiple comparisons test **(A, B, C, D, E, F, H, J)** and mixed-effects analysis with Šídák's multiple comparisons test **(G, I).** For males **(A, C, E, G, H)***n = 9* for NaChBac, and *n = 9* for all controls. For females **(B, D, F, I, J)***n* = 10 for NaChBac and *n* = 10 for controls. Data is represented as mean ± SEM, ⁎p < 0.05, ⁎⁎p < 0.01.

### 2.3 Metabolic parameters in chow fed mPFC-LH caspase and HFD fed mPFC-LH NaChBac

Given the previously mentioned metabolic changes in the chow-fed mPFC-LH^caspase^ and HFD-fed mPFC-LH^NaChBac^ group, we assessed various parameters in plasma, muscle, liver, and fat post-mortem as well as faeces. Plasma triglycerides and muscle glycogen were significantly higher in mPFC-LH^caspase^ males on a chow diet compared to control males and mPFC-LH^caspase^ females (Figure 5A,G). There were no significant differences in mPFC-LH^caspase^ muscle or liver triglycerides but a non-significant trend for an interaction of group x sex (two-way ANOVA, F (1, 24) = 3.197, p = 0.0864; Figure 5B,F). But there was a significant increase in muscle glycogen between mPFC-LH^caspase^ males and females, as well as a significant increase in the females compared to controls (two-way ANOVA main-effect of group F (1, 17) = 5.892, p = 0.0266; Fig. 5G). There was also a significant increase in male mPFC-LH^caspase^ liver glycogen compared to controls and mPFC-LH^caspase^ females (two-way ANOVA main-effect of sex F (1, 23) = 6.131, p = 0.0211; Fig. 5K). We saw no significant differences in plasma NEFA.

**Figure 5:**
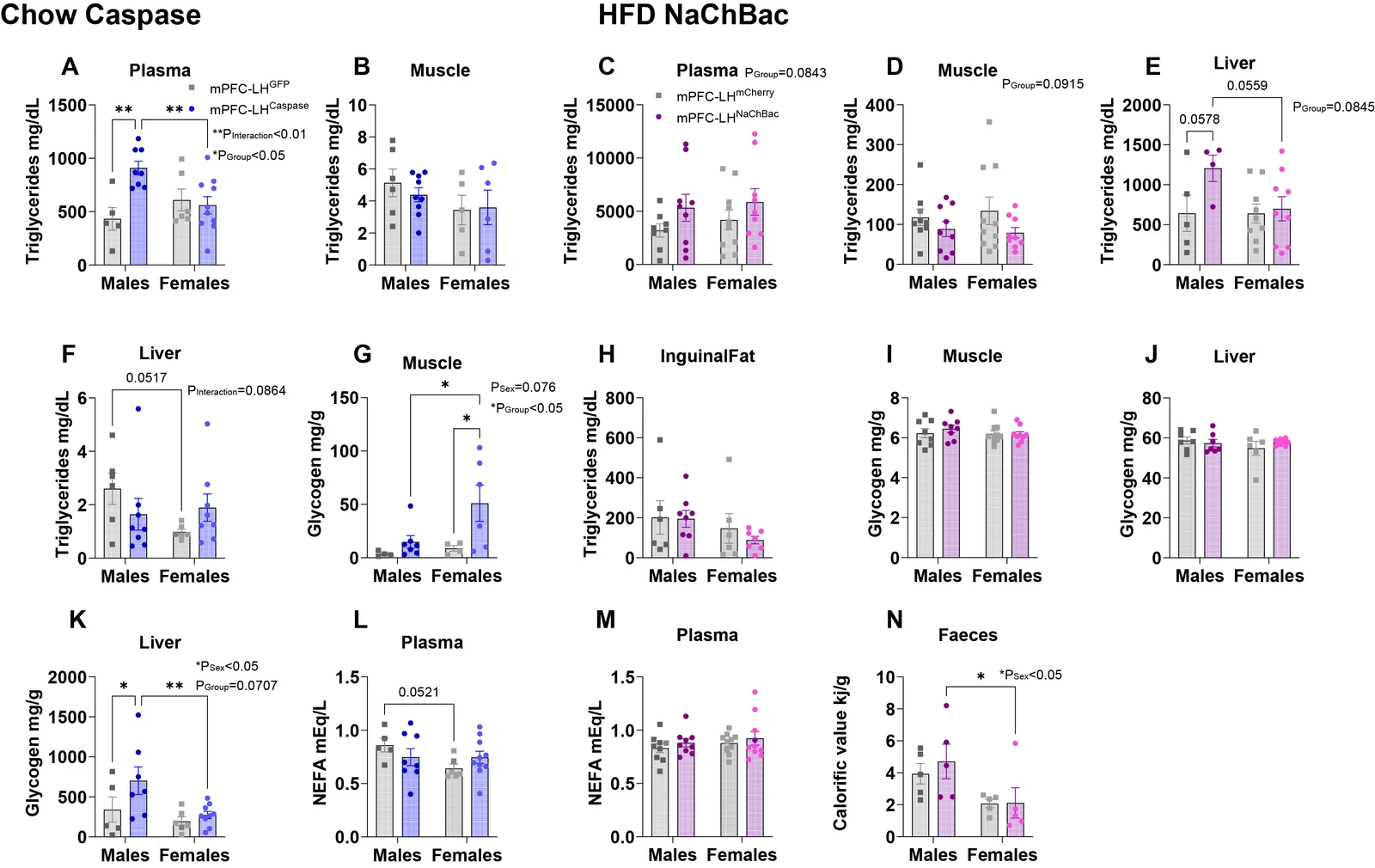
**Metabolic parameters from chow fed mPFC-LH caspase and HFD fed mPFC-LH NaChBac mice. (A)** Plasma triglycerides of male and female, control and caspase on chow. **(B)** Muscle triglycerides from quadricep of male and female, control and caspase on chow. **(C)** Plasma triglycerides of male and female, control and NaChBac on HFD. **(D)** Muscle triglycerides from quadricep of male and female, control and NaChBac on HFD. **(E)** Liver triglycerides of male and female, control and NaChBac on HFD. **(F)** Liver triglycerides of male and female, control and caspase on chow. **(G)** Muscle glycogen from quadricep of male and female, control and caspase on chow. **(H)** Inguinal fat triglycerides of male and female, control and NaChBac on HFD. **(I)** Muscle glycogen from quadricep of male and female, control and NaChBac on HFD. **(J)** Liver glycogen of male and female, control and NaChBac on HFD. **(K)** Liver glycogen of male and female, control and caspase on chow. **(L)** Plasma Non-esterified fatty acid (NEFA)s of male and female, control and caspase on chow. **(M)** Plasma NEFAs of male and female, control and NaChBac on HFD. **(N)** Calorific value of faeces of male and female, control and NaChBac on HFD. Two-way ANOVA with Uncorrected Fisher's LSD **(A, B, C, D, E, F, G, H, I, J, K, L, M, N)**. For chow fed control males **(A, K, L)**, *n* = 5, **(B, F)**, *n* = 6, **(G)**, *n* = 4 and females **(A, F, K, L)**, *n* = 6, **(B)**, *n* = 5, **(G)**, *n* = 4. For chow fed caspase males **(A, F, L)**, *n* = 8, **(B)**, *n* = 9, **(G, K)**, *n* = 7 and females **(A, L)**, *n* = 10, **(B, G)**, *n* = 6, **(F)**, *n* = 8, **(K)**, *n* = 9. For HFD fed control males **(C, I)**, *n* = 8, **(D, M)**, *n* = 9, **(E, N)**, *n* = 5, **(H)**, *n* = 6, **(J)**, *n* = 7 and females **(C, D, I, M)**, *n* = 10, **(E)**, *n* = 9, **(H, J)**, *n* = 6, **(N)**, *n* = 5. For HFD fed NaChBac males **(C, D, M)**, *n* = 9, **(E)**, *n* = 4, **(H, I)**, *n* = 8, **(J)**, *n* = 7, **(N)**, *n* = 5 and females **(C, D, E, I, J)**, *n* = 9, **(H)**, *n* = 7, **(M)**, *n* = 10, **(N)**, *n* = 5. Data is represented as mean ± SEM, ⁎p < 0.05, ⁎⁎p < 0.01.

For the HFD-fed mPFC-LH^NaChBac^ group there were no significant differences in plasma, muscle, or liver triglycerides, although there was a non-significant trend for a main-effect of group in all (Figure 5C,D, E). We also saw no significant differences in inguinal fat triglycerides, muscle or liver glycogen or NEFA (Figure 5H,I, J, M). But there was an effect of sex in the calorific value of faeces and a significant difference between mPFC-LH^NaChBac^ males and females (Fig. 5N).

### 2.4 Ablation of the mPFC-LH pathway alters stress-induced behaviour and hedonia in a diet- and sex-specific manner

Previous studies have demonstrated that stress activates mPFC-LH neurons, whereas activation of mPFC-LH neurons drives changes in social and aggressive behaviour [26], [27], [28]. To assess if mPFC-LH caspase ablation affects exploration and anxiety in response to stress, we placed mPFC-LH^Caspase^ mice or controls in an open-field test with and without a 15-minute restraint tube stress (Figure 6A). Although restraint stress increased plasma corticosterone 15 min after stress induction, there were no significant differences between mPFC-LH^Caspase^ mice or controls in males or females on chow or HFD at baseline, 15 min or 2 h after stress (Figure 6B,C, D, E). These results suggest mPFC-LH ablation has not impacted the Hypothalamic-Pituitary Adrenal (HPA) axis. To aid in the interpretation of the behavioural phenotype we have created an exploration index encompassing open field distance moved, inner zone % and rearing duration and found that mPFC-LH ablation in chow, but not HFD, fed males resulted in less open field exploration after stress (two-way ANOVA; main effect of stress condition (F (1, 13) = 41.06, p < 0.0001) and condition × group interaction (F (1, 13) = 8.199, p = 0.0133); Figure 6F,L). This index examines exploration more generally and all findings are in line with the individual variables found in the supplemental figures. Aspects of this exploration index included less movement after stress in chow fed mPFC-LH^Caspase^ male mice compared to controls (Supp. Fig. 6A). There were no significant differences in the exploration index in chow fed mPFC-LH^Caspase^ female mice (Fig. 6G), although they reared less in the open field following stress (Supp. Fig. 6K). HFD-fed mPFC-LH^Caspase^ female mice had a non-significant trend for increased exploration after stress (Fig. 6M), presumably due to increased movement following restraint stress compared to controls (Supp. Fig. 7H). We also created an anxiety index incorporating the inverse of inner zone % and grooming duration in an open-field and found no significant differences in anxiety in males on either a chow or HFD (Figure 6H,N). Although mPFC-LH^Caspase^ female mice on a chow diet showed no significant differences in anxiety (Fig. 6I), HFD-fed mPFC-LH^Caspase^ female mice had a significantly lower anxiety index after stress compared to controls (two-way ANOVA main effect for stress condition (F (1, 13) = 7.341, p = 0.0179), group (F (1, 13) = 7.794, p = 0.0153), and a significant interaction (F (1, 13) = 7.186, p = 0.0189); Fig. 6O), partially due to less grooming after stress compared to controls (Supp. Fig. 7J).

**Figure 6:**
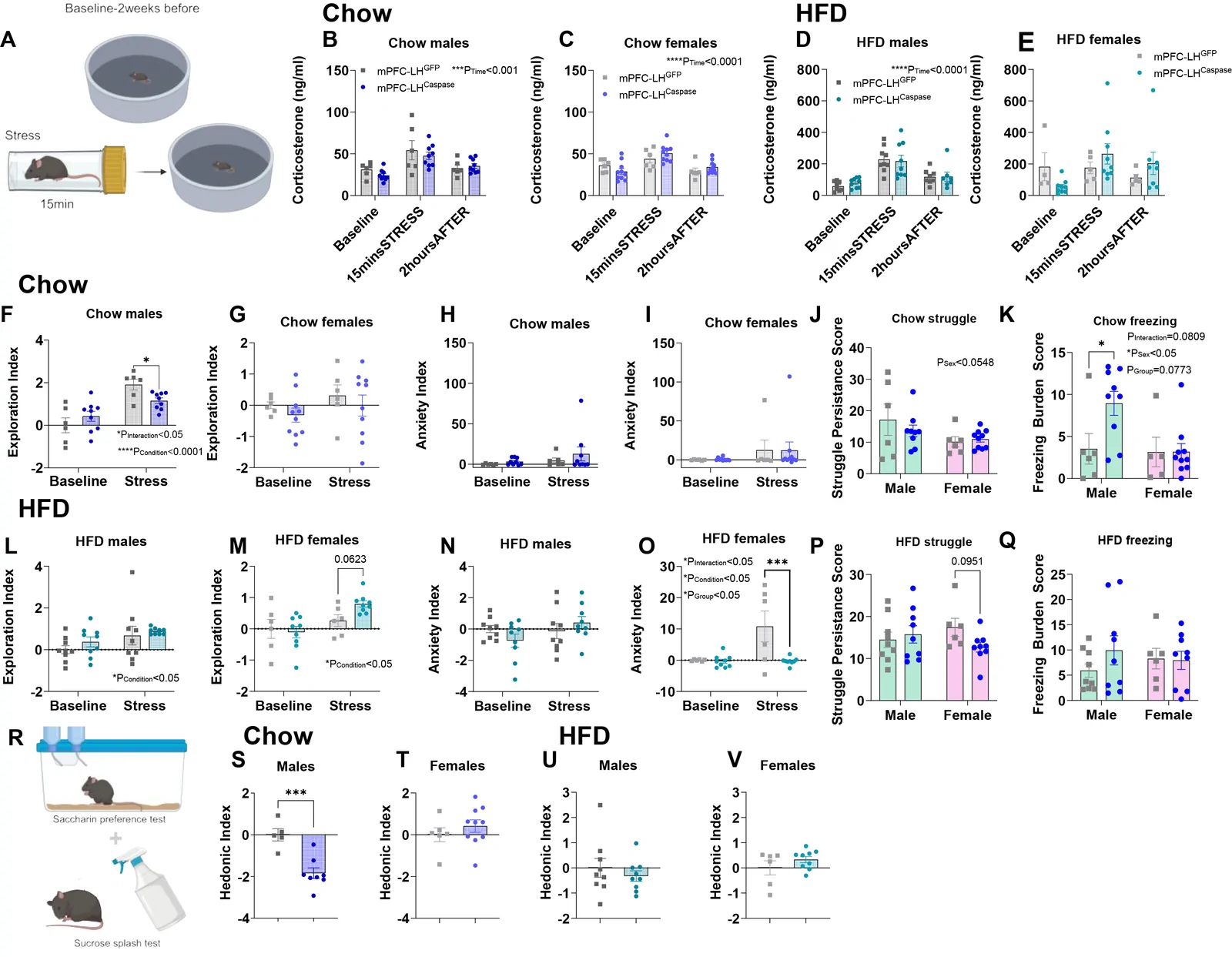
**mPFC-LH caspase males on a chow diet explore less after stress and freeze more, whilst females on HFD are less anxious after stress. (A)** Schematic of baseline open-field test and 15-min restraint tube stress then open-field test. **(B)** Corticosterone of baseline, 15-min restraint tube stress, and 2 h after in chow fed controls and caspase males. **(C)** Corticosterone of baseline, 15-min restraint tube stress, and 2 h after in chow fed controls and caspase females. **(D)** Corticosterone of baseline, 15-- restraint tube stress, and 2 h after in HFD fed controls and caspase males. **(E)** Corticosterone of baseline, 15-min restraint tube stress, and 2 h after in HFD fed controls and caspase females. **(F)** Exploration index of baseline and after stress in the open-field for chow fed controls and caspase males. **(G)** Exploration index of baseline and after stress in the open-field for chow fed controls and caspase females. **(H)** Anxiety index of baseline and after stress in the open-field for chow fed controls and caspase males. **(I)** Anxiety index of baseline and after stress in the open-field for chow fed controls and caspase females. **(J)** Struggle persistence score of male and female control and caspase mice during the restraint tube stress fed a chow diet. **(K)** Freezing burden score of male and female control and caspase mice during the restraint tube stress fed a chow diet. **(L)** Exploration index of baseline and after stress in the open-field for HFD fed controls and caspase males. **(M)** Exploration index of baseline and after stress in the open-field for HFD fed controls and caspase females. **(N)** Anxiety index of baseline and after stress in the open-field for HFD fed controls and caspase males. **(O)** Anxiety index of baseline and after stress in the open-field for HFD fed controls and caspase females. **(P)** Struggle persistence score of male and female control and caspase mice during the restraint tube stress fed HFD. **(Q)** Freezing burden score of male and female control and caspase mice during the restraint tube stress fed HFD. **(R)** Schematic showing the saccharin preference test and sucrose splash test used to measure hedonia. **(S)** Hedonic index of male controls and caspase on chow. **(T)** Hedonic index of female controls and caspase on chow. **(U)** Hedonic index of male controls and caspase on HFD. **(V)** Hedonic index of female controls and caspase on HFD. For males' corticosterone on chow *n* = 6 for controls and *n* = 9 for caspase; for females *n* = 6 for controls and *n* = 10 for caspase with two-way repeated-measures ANOVA with Tukey's multiple comparisons test **(B, C)**. For males' corticosterone on HFD *n* = 9 for controls and *n* = 9 for caspase; for females *n* = 6 for controls and *n* = 9 for caspase with mixed-effects analysis with Tukey's multiple comparison test **(D, E)**. For males' exploration and anxiety on chow *n* = 6 for controls and *n* = 9 for caspase; for females *n* = 6 for controls and *n* = 10 for caspase with two-way repeated-measures ANOVA with Uncorrected Fisher's LSD **(F, G, H, I)**. For males' exploration and anxiety on HFD *n* = 9 for controls and *n* = 9 for caspase; for females *n* = 6 for controls and *n* = 9 for caspase with two-way repeated-measures ANOVA with Uncorrected Fisher's LSD **(L, M, N, O)**. Two-way ANOVA with Uncorrected Fisher's LSD **(J, K, P, Q)**. For hedonic index of caspase males on chow *n* = 5 for controls and *n* = 8 for caspase; for females *n* = 6 for controls and *n* = 10 for caspase. For hedonic index of caspase males on HFD *n* = 9 for controls and *n* = 9 for caspase; for females *n* = 6 for controls and *n* = 9 for caspase. Unpaired t-test **(S, U)**, and Mann Whitney test **(T, V)**. Data is represented as mean ± SEM, ⁎p < 0.05, ⁎⁎⁎p < 0.001.

The exploration and anxiety indices assess behaviour in an open field after restraint stress, however they cannot be used to assess behaviour during restraint stress. To assess whether mPFC-LH^Caspase^ ablation affected behaviour during restraint stress we used DeepLabCut to determine struggle and freezing by deriving a struggle persistence score and freezing burden score; as these are core characteristics of the ‘fight or flight’ stress response [29], [30]. Chow fed males in both groups had a trend for a higher struggle persistence than chow fed female mice (two-way ANOVA main effect of sex F (1, 27) = 4.032, p = 0.0548; Fig. 6J). In addition, chow fed mPFC-LH^Caspase^ male mice, which exhibited a lower exploration index, also had a significantly higher freezing burden during restraint (Fig. 6P), suggesting freezing during restraint predicted reduced exploration levels following restraint stress. In HFD mice, there was a non-significant trend for mPFC-LH^Caspase^ female mice to struggle less than controls (Fig. 6P) and no significant difference in their freezing burden scores (Fig. 6Q).

We next examined whether mPFC-LH ablation affected hedonic processing. We created a hedonic index by integrating average preference and grooming duration associated with a saccharin preference test and sucrose splash test. We found mPFC-LH^Caspase^ male mice on a chow diet had a significantly lower hedonic index than controls (Fig 6S). No differences observed in mPFC-LH^Caspase^ females compared to controls (Fig 6T). This is shown by a significant difference between groups in a saccharin preference test and significantly lower grooming compared to controls during a sucrose splash test in males (Supp. Fig. 8A and C).

### 2.5 Activation of the mPFC-LH pathway alters stress-induced behaviour and hedonia in a diet- and sex-specific manner

To determine how chronic activation of mPFC-LH neurons affects stress-induced behaviour, we examined stress-induced plasma CORT, exploration and anxiety in an open field and struggle persistence and freezing burden during restraint. Although plasma CORT was not significantly different during restraint stress, it was significantly reduced in chow-fed mPFC-LH^NaChBac^ male mice 2 h after restraint stress (two-way ANOVA, main effect for time (F (1.692, 28.76) = 18.92, p < 0.0001), group (F (1, 17) = 7.850, p = 0.0123), and interaction (F (1.692, 28.76) = 6.034, p = 0.009); Figure 7B). A similar trend was observed in HFD-fed mPFC-LH^NaChBac^ male mice compared to controls, however, this did not reach statistical significance (Fig. 7C). mPFC-LH^NaChBac^ female mice fed either chow or HFD exhibit no significant differences in plasma CORT at baseline, during or after restraint stress (Figure 7D,E).

**Figure 7:**
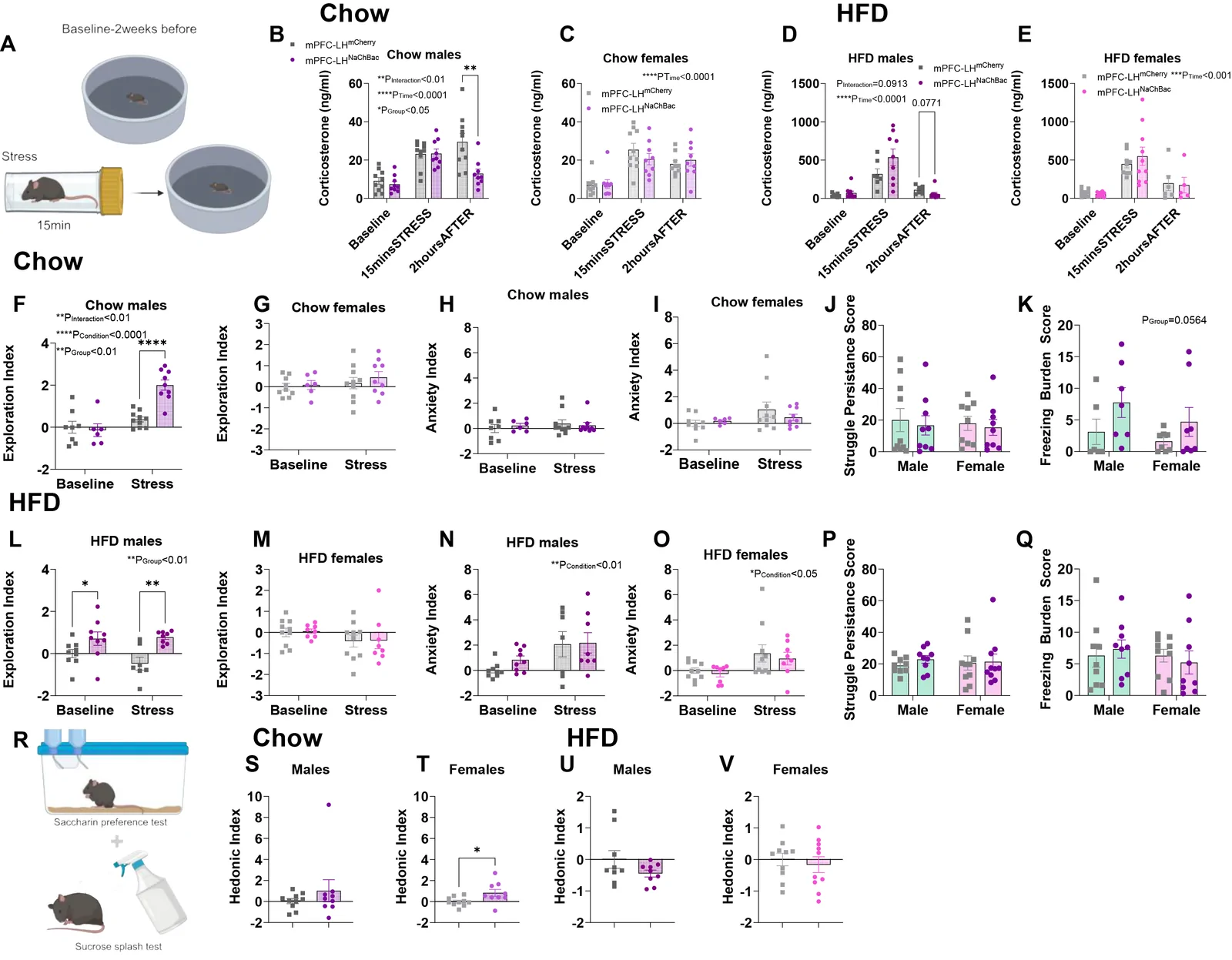
**mPFC-LH NaChBac males on a chow diet have lower corticosterone after stress and are more explorative regardless of diet. (A)** Schematic of baseline open-field test and 15-min restraint tube stress then open-field test. **(B)** Corticosterone of baseline, 15-min restraint tube stress, and 2 h after in chow fed controls and NaChBac males. **(C)** Corticosterone of baseline, 15-min restraint tube stress, and 2 h after in chow fed controls and NaChBac females. **(D)** Corticosterone of baseline, 15-min restraint tube stress, and 2 h after in HFD fed controls and NaChBac males. **(E)** Corticosterone of baseline, 15-min restraint tube stress, and 2 h after in HFD fed controls and NaChBac females. **(F)** Exploration index of baseline and after stress in the open-field for chow fed controls and NaChBac males. **(G)** Exploration index of baseline and after stress in the open-field for chow fed controls and NaChBac females. **(H)** Anxiety index of baseline and after stress in the open-field for chow fed controls and NaChBac males. **(I)** Anxiety index of baseline and after stress in the open-field for chow fed controls and NaChBac females. **(J)** Struggle persistence score of male and female control and NaChBac mice during the restraint tube stress fed a chow diet. **(K)** Freezing burden score of male and female control and NaChBac mice during the restraint tube stress fed a chow diet. **(L)** Exploration index of baseline and after stress in the open-field for HFD fed controls and NaChBac males. **(M)** Exploration index of baseline and after stress in the open-field for HFD fed controls and NaChBac females. **(N)** Anxiety index of baseline and after stress in the open-field for HFD fed controls and NaChBac males. **(O)** Anxiety index of baseline and after stress in the open-field for HFD fed controls and NaChBac females. **(P)** Struggle persistence score of male and female control and NaChBac mice during the restraint tube stress fed HFD. **(Q)** Freezing burden score of male and female control and NaChBac mice during the restraint tube stress fed HFD. **(R)** Schematic showing the saccharin preference test and sucrose splash test used to measure hedonia. **(S)** Hedonic index of male controls and NaChBac on chow. **(T)** Hedonic index of female controls and NaChBac on chow. **(U)** Hedonic index of male controls and NaChBac on HFD. **(V)** Hedonic index of female controls and NaChBac on HFD. For males' corticosterone on chow *n* = 10 for controls and *n* = 9 for NaChBac; for females *n* = 10 for controls and *n* = 9 for NaChBac with two-way repeated-measures ANOVA with Tukey's multiple comparisons test **(B)**, and mixed-effects analysis with Tukey's multiple comparisons test **(C)**. For males' corticosterone on HFD *n* = 8 for controls and *n* = 9 for NaChBac; for females *n* = 10 for controls and *n* = 10 for NaChBac with mixed-effects analysis with Tukey's multiple comparison test **(D, E)**. For males' exploration and anxiety on chow *n* = 10 for controls and *n* = 9 for NaChBac; for females *n* = 10 for controls and *n* = 9 for NaChBac with mixed-effects analysis with Uncorrected Fisher's LSD **(F, G, H, I)**. For males' exploration and anxiety on HFD *n* = 9 for controls and *n* = 9 for NaChBac; for females *n* = 10 for controls and *n* = 9 for NaChBac with mixed-effects analysis with Uncorrected Fisher's LSD **(L, M, N, O)**. Two-way ANOVA with Uncorrected Fisher's LSD **(J, K, P, Q)**. For hedonic index of NaChBac males on chow *n* = 10 for controls and *n* = 9 for NaChBac; for females *n* = 10 for controls and *n* = 9 for NaChBac. For hedonic index of NaChBac males on HFD *n* = 9 for controls and *n* = 9 for NaChBac; for females *n* = 10 for controls and *n* = 10 for NaChBac. Unpaired t-test **(V)**, Mann Whitney test **(S, U)**, Welch's t-test **(T)**. Data is represented as mean ± SEM, ⁎p < 0.05, ⁎⁎p < 0.01, ⁎⁎p < 0.001, ⁎⁎⁎⁎p < 0.0001.

Interestingly, mPFC-LH^NaChBac^ male mice on a chow diet had a significantly higher exploration index after restraint stress compared to controls (mixed-effects analysis, main effects for condition (F (1, 12) = 36.79, p < 0.0001), group (F (1, 17) = 8.926, p = 0.0083), and interaction (F (1, 12) = 17.67, p = 0.0012); Fig. 7F). Even though there was no difference between groups in any parameters of the exploration index individually (Supp. Fig. 9). On HFD, mPFC-LH^NaChBac^ male mice had a significantly higher exploration index for both at baseline and after restraint stress (mixed-effects analysis, main effects for group (F (1, 16) = 13.87, p = 0.0018); Fig. 7L), suggesting chronic activation of mPFC-LH provides resilience to stress-induced events. There were no significant differences in the exploration index for mPFC-LH^NaChBac^ female mice on either chow or HFD (Figure 7G,M). mPFC-LH^NaChBac^ expression had no effect on anxiety in males and females on chow diet (Figure 7H,I) or HFD (Figure 7N,O). HFD fed male and female mice exhibited elevated anxiety following restraint stress regardless of mPFC-LH^NaChBac^ expression (Figure 7N,O). This resulted from an increase in grooming duration when stressed compared to non-stressed in mPFC-LH^NaChBac^ males and a non-significant trend towards an increase in grooming when stressed regardless of group (Supp. Fig. 10C and J). During restraint stress, we did not detect any effect of mPFC-LH^NaChBac^ activation on the struggle persistence score or freezing burden score in males or females on chow or HFD (Figure 7J,K, P, Q).

Chow fed mPFC-LH^NaChBac^ females, but not males, had a significantly higher hedonic index than controls (Figure 7S,T) and no differences were observed in HFD fed males or females (Figure 7U,V). This is demonstrated by a significant increase in the amount of saccharin consumed and grooming after the splash test in chow fed mPFC-LH^NaChBac^ females compared to controls (Supp. Fig. 11E and F). These results suggest mPFC-LH ablation reduces hedonic processing in chow males only, whereas chronic mPFC-LH activation increased hedonia in chow fed females only, highlighting both diet- and sex-specific effects.

### 2.6 Active transcriptomic expression of male and female mPFC-LH neurons

To assess potential transcriptomic differences between mPFC-LH neurons in males and females, we used Translating ribosomal affinity purification (TRAP), which allows for pathway specific analysis of actively translated mRNAs of mPFC-LH neurons. Out of 16,874 genes, we observed 34 genes differentially expressed (log2fold change >1, adjusted p-value <0.05; Figure 8A). To confirm our samples were neurons we looked for common markers of neurons including proteins, transporters, and receptors (Supp RNA_data). We found several that were not significantly differentially expressed (Fig 8C). Out of the 34 genes that were significantly differentially expressed, 6 were protein coding mRNAs, 11 microRNAs, and 17 we have categorised as other (long or small non-coding RNAs) (Fig 8B). From the 6 protein coding mRNAs, 4 (Myh8, Epb41, Stx3, Pde5a) were decreased and 2 (Ccnb1ip1, Cpne9) increased in males compared to females (Fig 8D). MicroRNA target analysis reveals 3 of these mRNAs (Pde5a, Epb41, Stx3) to be likely targets of differentially expressed microRNAs (Fig 8E). Pathway enrichment analysis using KEGG of mRNAs reveals no statistically significant pathways, possibly due to low number of genes, whilst pathway enrichment for microRNAs reveals 57 pathways statistically significant (Supp RNA_data). Heat maps of normalized gene counts of all groups shows consistent levels amongst groups except for Ccnb1ip1 upregulation in males seems to be driven by 2 samples (Fig 8B).

**Figure 8:**
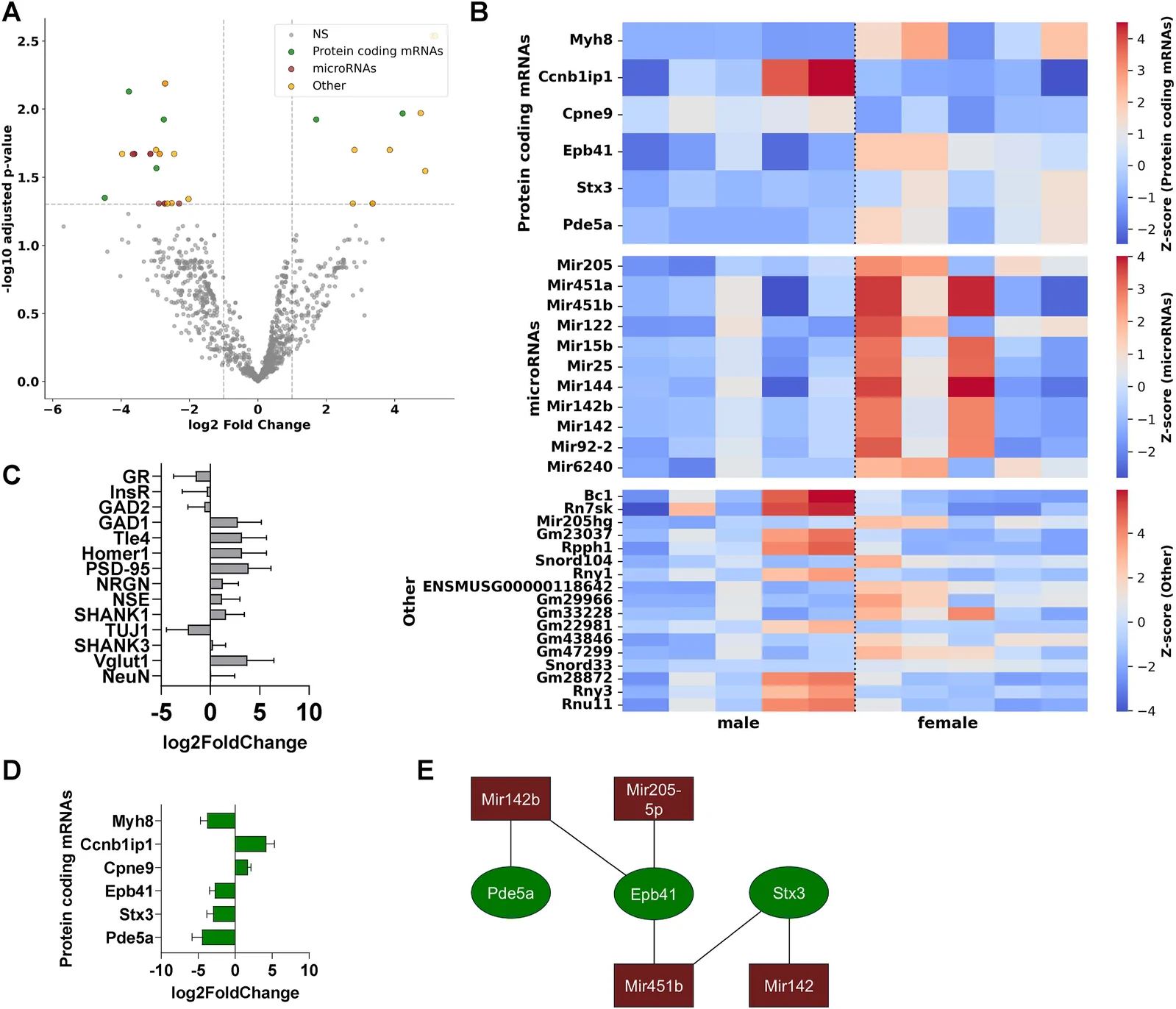
**TRAP-seq of male and female mPFC-LH neurons.(A)** Volcano plot showing 34 genes differentially expressed in male vs female mPFC-LH neurons including 6 protein-coding mRNAs, 11 microRNAs, and other genes including small and long non-coding RNAs (log2foldchange >1, with adjusted p-value <0.05). **(B)** Grouped heat map of normalized gene counts for each sample of significantly differentially expressed genes. **(C)** log2foldchange of neuronal marker genes to confirm cell type of samples that were not significant for differential expression. **(D)** log2foldchange of significant differentially expressed protein coding mRNAs with log2foldchange standard error. **(E)** Visual interaction network of significantly differentially expressed protein coding mRNAs likely binding of microRNAs using TargetScan. n = 5 for males and females.

## 3 Discussion

These studies were conducted to explore the long-term impact of mPFC-LH ablation or activation on metabolic and behavioural outcomes. We show that chronic ablation or activation of the mPFC-LH pathway via caspase-mediated apoptosis or NaChBac-mediated activation resulted in sex- and diet-dependent effects on metabolism and behaviour. mPFC-LH ablation increased body weight in males, but not females, on a chow diet, and caused no differences in body weight on HFD-fed males or females, suggesting the high energy content of the HFD overwhelmed the subtle body weight differences on a chow diet. mPFC-LH ablation reduced exploration, hedonia and elevated freezing in chow-fed, but not HFD-fed males, whereas mPFC-LH ablation in females reduced anxiety in HFD-fed mice. In contrast, chronic mPFC-LH activation with NaChBac reduced body weight gain in female mice only on a HFD and modestly increased hedonia in chow fed female mice. Despite having no effect on body weight on either chow or HFD, chronic mPFC-LH activation in males lowered corticosterone levels and increased exploration immediately after stress. These results indicate chronic mPFC-LH ablation impaired, whereas chronic mPFC-LH activation, amplified the appropriate behavioural response to stress, although sex-specific and diet-specific effects were observed. In general, our studies reveal that the cortico-hypothalamic pathway modestly affects energy homeostasis and promotes behavioural resistance to acute stress, although the downstream target populations within the LH remain to be determined.

Previous studies have demonstrated that acute stressors and anxiety-inducing stimuli [31] activate the mPFC-LH pathway, with stimulation of this pathway causes anxiety-like responses and suppression of feeding [17], suggesting long term ablation or activation may have effects on body weight and metabolism. Interestingly chronic mPFC-LH ablation using caspase increased body weight only in chow fed males and the higher weight gain is predominantly ascribed to an increased gain in lean mass. Moreover, despite no difference in body weight, mPFC-LH ablation in chow fed female mice also increased lean mass gain. Although the mechanisms responsible are unknown, a recent study demonstrated *slc12a8*, a nicotinamide mononucleotide transporter, in the LH maintained skeletal muscle mass via the sympathetic nervous system [32]. In addition, overexpression of *slc12a8* specifically in the LH improved sarcopenia and frailty measures in aged mice [32]. Thus, it is possible that mPFC-LH ablation promotes *slc12a8* expression in the LH and thereby increases lean muscle mass. We did not explore the mechanisms promoting muscle mass within the muscle itself, although we did observe increased muscle glycogen, which is known to amplify mTORC1 signalling and promote protein synthesis [33], [34], [35]. This suggests mPFC-LH ablation may increase mTORC1 signalling in muscle, although this hypothesis requires experimental validation. Furthermore, mPFC-LH ablation in chow fed mice increased glucose clearance in males and insulin sensitivity in females, effects likely mediated by a gain in lean muscle mass [36]. Interestingly, the increased lean mass gain in mPFC-LH ablated females was not accompanied with an increase in bodyweight or fat mass gain compared to controls. This is likely due to limitations in our body composition analysis, such as inherent sampling variation and lack of bone density measurements or differences in organ mass. In addition, mPFC-LH ablation in chow-fed males improved glucose clearance despite elevated fasting blood glucose. These mice also displayed an increase in plasma triglycerides, which may underlie the observed effects on fasting blood glucose [37] via glycerol-derived gluconeogenesis [38], and liver glycogen, which may mediate glucose clearance through increased hepatic glucose uptake and storage [39].

We used NaChBac to examine chronic mPFC-LH activation on weight gain, as this strategy has previously been used to promote weight gain by chronically activating GABA or non-leptin receptor neurons in the arcuate nucleus of the hypothalamus [25], [40]. Despite increasing the firing properties of mPFC neurons projecting to the LH, chronic activation with NaChBac had no effect on body weight in either sex on a chow diet, but decreased weight gain and improved glucose clearance and insulin sensitivity in HFD fed females only. We cannot rule out differences in intrinsic electrophysiological properties between sexes in LH-projecting mPFC neurons due to our small sample size.

Collectively, chronic mPFC-LH manipulation with either caspase or NaChBac produced reciprocal changes in body weight, albeit in a sex- and diet-specific manner, where caspase ablation increases weight gain in males on a chow diet and NaChBac activation decreases body weight in HFD-fed females. The effects on body weight are consistent with the actions of LH neurons, as both LH^Vglut2^ and LH^Vgat^ neurons regulate body weight [15], [16]. In these earlier studies, ablation of LH^Vgat^ neurons restricts body weight gain on a calorie dense diet [15] and ablation of LH^Vglut2^ neurons promotes weight gain on a calorie-dense diet [16]. Consistent with results presented in our studies, ablation of LH^Vglut2^ neurons produced diet-specific effects, where weight gain only differed on a calorie-dense diet and not on standard chow [16]. In addition, activation of LH^LepR^ neurons also decreased body weight over a 3-day period on a chow diet [41]. Thus, diet-specific changes in body weight, following mPFC-LH manipulation, are consistent with the effect of LH neurons on body weight. Unfortunately, these earlier studies only examined male mice, making it impossible to compare sex-specific effects with the existing literature. Current studies show that mPFC neurons target LH^Vglut2^, LH^Vgat^, LH^LepR^ neurons or LH^Orexin^ neurons [31], [42]. These results highlight the importance of understanding the downstream LH neuronal populations targeted by mPFC neurons. Indeed, the diet- or sex-specific differences we observed with chronic ablation compared to chronic activation may be due to differential targeting of LH populations in males and females. Future studies are required to investigate this possibility.

The function of the mPFC-LH pathway in response to stressful stimuli [17] has most often been ascribed to social, anxiety-like and aggressive behaviour. Indeed, the mPFC-LH pathway controls reward-based social behaviour [26], increases anxiety-like behaviour [31] as well as increasing or decreasing aggression [27], depending on the pathway recruited [28]. However, these studies all examined behaviour in response to acute chemogenetic or optogenetic manipulation. In contrast, our studies examined whether chronic activation or ablation affected stress-induced behaviour. Chronic mPFC-LH ablation suppressed open field exploration following acute stress, increased freezing behaviour and reduced hedonia in chow fed mice, whereas chronic NaChBac activation caused reciprocal increases in exploration and decreased plasma CORT 2 h after acute stress. This suggests the mPFC-LH pathway promotes an adaptive response that increases resilience to stress, at least in male mice. Thus, the acute stress-induced increase in mPFC-LH activity, as previously observed [17], [31] presumably represents an important functional component of a neural network that promotes behavioural resilience.

In the long term, the inability of the mPFC-LH pathway to respond appropriately to stress, as seen with chronic ablation in males, causes anhedonia, freezing and reduced exploration during and following stress. Anhedonia is a hallmark of a variety of mental disorders, such as major depressive disorder [43], [44], highlighting that our observations are consistent with the known role for the mPFC in stress-induced depression [45], [46], [47], at least in males. Intriguingly, chronic activation of the mPFC-LH pathway in females increased hedonic responses but had no effect on stress-induced exploration following either ablation or activation. In fact, chronic ablation of the mPFC-LH pathway reduced stress-induced anxiety in HFD-fed females. While the mechanisms responsible for these sex differences are unknown, there are numerous reports of sex differences linked to stress and mPFC function [48], [49], [50], [51]. Taken together, our observations suggest the mPFC-LH pathway may play an under-appreciated role in the sex-specific pathogenesis of depression and potentially other stress-related disorders [46], [49], [52], [53].

The active transcriptomic differences within mPFC-LH neurons in males and females included microRNAs, noncoding RNAs and protein coding mRNAs. 6 differentially expressed protein coding mRNAs included 1) Myh8, a marker for mouse cortex layer 5 somatostatin interneurons [54], [55]; 2) Epb41, a regulator of neuronal morphology and cytoskeletal interaction [56] and implicated in pain [57]; 3) Stx3, a regulator of synaptic plasticity and receptor trafficking to the postsynaptic membrane [58], implicated in novelty preference in the hippocampus [59]; 4) Pde5a, a regulator of cyclic GMP and protein kinase G [60], implicated in chronic stress [61] and neuropsychiatric conditions [60], [62], [63]; 5) Ccnb1ip1, involved in neural development, learning, memory and stress responsiveness [64], [65]; 6) Cpne9, a calcium dependent phospholipid binding protein involved in intracellular signalling of a subtype of mPFC excitatory neurons [66], [67], also sensitive to stress [68]. In addition, we observed vglut1 expression, although not differentially expressed in males and females, confirming the previously described glutamatergic nature of the mPFC-LH circuit [17]. Thus, our active transcriptomic changes are consistent with known roles for mPFC neurons in stress, learning and memory, and suggest subtle sex differences in mPFC-LH neurons are linked to genes involved in synaptic plasticity and intracellular signalling.

An important area for future investigation is to determine how the mPFC-LH pathway integrates different types of stress with feeding behaviour, given that stress can promote the excessive intake of highly palatable foods and contribute to obesity [42], [69], [70]. Indeed, the mPFC is implicated in stress-induced food intake, including in patients with compulsive eating disorders, such as binge eating disorder or bulimia nervosa [71], [72], [73]. That said, studies on the mPFC-LH pathway and feeding have produced conflicting results, with acute activation of the pathway suppressing food intake [17], whereas social stress only causes increases in palatable food intake [42]. Moreover, cue-potentiated stimulation of the mPFC-LH^orexin^ pathway leads to overconsumption of high-fat diet [74]. In our model of chronic ablation or activation of the mPFC-LH pathway we found no differences in food intake, although we measured food intake 20 weeks after surgery once mice were singly housed for the purpose of conducting behavioural experiments. Due to the diverse role, and heterogeneity, of LH neuronal populations, LH outputs to other brain regions, both within and outside the hypothalamus, are also likely to contribute to differences in behaviour [75]. For example, the LH^Glutamate^ circuit regulates dopamine function in the ventral tegmental area [76] and enhances stress-driven fat intake.

In conclusion, we show that chronic manipulation of the mPFC-LH pathway via caspase-mediated deletion or NaChBac-induced excitation causes sex and diet-specific metabolic and behavioural differences. Further studies will be required to investigate the mechanisms of action of these effects to gain further insight into how these areas control body weight and behaviour, ultimately to inform treatment strategies for obesity and mental disorders.

## 4 Methods

### 4.1 Animals

All experiments were conducted in accordance with Monash University Animal Ethics Committee and were housed in Monash Animal Research Platform laboratories. All mice were obtained from the Monash Animal Research Platform (MARP; Clayton, VIC, Australia; line sourced from JAX®) and housed under standard laboratory conditions at 23 °C with a relative humidity of 50–70% in a 12hr light/dark cycle and provided with ad libitum access to chow food and water (20% protein 4.8% fat chow diet; Specialty Feeds, Western Australia) or high fat diet (SF01-001, 20.9% protein 23.5% fat diet, Specialty Feeds, Western Australia). All mice were C57BL/6 J 8–10 weeks at time of surgery and fs-Targeted Recombination in Active Populations mice were used for TRAP (Translating Ribosome Affinity Purification) RNA sequencing (B6.129S4-Gt (ROSA)26Sor^tm1(CAG−EGFP/Rpl10a,−birA)Wtp^/J; stock number 022367). In the absence of cre recombinase, fs-TRAP express a floxed EGFP-L10a ribosome tag in an untranslated conformation. The presence of cre recombinase drives expression of EGFP and therefore serves to mark neurons expressing cre recombinase. A total of 154 mice were used (50% male) and all animals were randomly assigned to each group. Within each experiment (caspase/controls; NaChBac/controls) mice were from the same litter.

### 4.2 Stereotaxic surgery

Mice were anaesthetized using isoflurane at a concentration of 5% for induction followed by 1–3% for maintenance. Anesthesia was indicated by loss of pedal withdrawal reflex and mice were placed in a heated (37 °C) stereotaxic frame (Stoelting), then subcutaneously injected with Metacam (5 mg/kg, Boehringer-Ingelheim). Prior to an incision being made in the centre of the skull to expose bregma, Lacri-Lube (Allergan) was applied to the eyes to prevent corneal damage. Adeno-associated virus injections were done using a 5 μl Hamilton Syringe Neuros 75 N from skull surface with y coordinate from bregma. Within the same surgery a cre-dependent retrograde virus (24593-AAVrg AAV-pgk-Cre (Addgene)) was injected bilaterally into the LH (coordinates −1.3/±1/-4.9) for all experiments (caspase validation + RNAseq + caspase/NaChBac). Subsequently either a cre-dependent caspase or NaChBac (pAAV-flex-taCasp3-TEVp (AAV5-45580; addgene); PT-2965 rAAV-EF1a-DIO-NaChBac-P2A-EGFP-WPREs, AAV2/9 (BrainVTA)) virus was injected bilaterally into the mPFC (coordinates +1.6/±0.3/-1.8) for experimental mice or control (pAAV-hSyn-DIO-EGFP (AAV5 – 50,457 (Addgene); pAAV-hSyn-DIO-mCherry (AAV5 – 50,459; Addgene) (caspase/NaChBac experiments). 200 nl of undiluted virus was used for each injection with 4 min between injections. Lastly, mice were sutured and placed on a heated cage for recovery. Mice had at least 2 weeks of recovery before the commencement of experiments.

### 4.3 Electrophysiology

Mice were deeply anesthetized with isoflurane (3% in 100% O_2_) and transcardially perfused with approximately 40 ml ice-cold cutting artificial cerebrospinal fluid (ACSF), continuously bubbled with carbogen (95% O_2_, 5% CO_2_). The cutting ACSF contained (in mM): choline chloride 110, NaHCO_3_ 26, NaH_2_PO_4_·H_2_O 1.25, KCl 2.5, sodium ascorbate 11.6, sodium pyruvate 3.1, MgCl_2_·6H_2_O 7, CaCl_2_·2H_2_O 0.5, glucose 10. The brain was then rapidly removed, placed in carbogenated ice-cold cutting ACSF, and coronal slices (400 μm) containing the medial prefrontal cortex (mPFC) were prepared using a vibratome (VT1000S, Leica). Slices were incubated in warmed incubation ACSF at 36 °C for 30–40 min then maintained at room temperature for at least 30 min before recording. The incubation ACSF contained (in mM): NaCl 92, NaHCO_3_ 30, NaH_2_PO_4_·H_2_O 1.2, KCl 2.5, HEPES 20, sodium pyruvate 3, MgCl_2_·6H_2_O 2, CaCl_2_·2H_2_O 2, glucose 25.

Whole-cell current-clamp recordings were performed using infrared differential interference contrast optics on an upright microscope (BX51, Olympus). Fluorescently labelled neurons in the mPFC expressing either EGFP or mCherry were visually identified and targeted for recording. During recording slices were continuously perfused (5 ml/min) with carbogenated recording ACSF containing (in mM): NaCl 125, NaHCO_3_ 25, NaH_2_PO_4_·H_2_O 1.25, KCl 3, MgCl_2_·6H_2_O 1, CaCl_2_·2H_2_O 2, glucose 25, maintained at 32–35 °C using an in-line temperature controller. Patch pipettes were prepared using a micropipette puller (P-97; Sutter) and filled with a K-gluconate-based intracellular solution containing (in mM): K-gluconate 130, KCl 10, HEPES 10, MgATP 4, NaGTP 0.3, Na-phosphocreatine 10. Patch pipettes had open tip resistances of 4–7 MΩ (thick-walled borosilicate glass). Biocytin (0.5% w/v) was included in the internal solution for post hoc morphological reconstruction. Voltage signals were recorded using a current-clamp amplifier (BVC-700 A; Dagan), low-pass filtered at 10 kHz, and digitised at 50 kHz (ITC-18, Instrutech) using AxoGraph X software (Axograph Scientific). After break-in, series resistance and pipette capacitance were compensated in current-clamp using bridge balance and capacitance neutralization and monitored throughout recordings. Intrinsic membrane properties and excitability were characterized using 1 s hyperpolarizing and depolarizing current steps.

Following recording, slices containing biocytin-filled neurons were fixed in 4% paraformaldehyde in phosphate-buffered saline (PBS) for 2 h at room temperature and then overnight at 4 °C. After washing in PBS (3 × 10 min), slices were permeabilised and blocked for 1 h at room temperature in PBS containing 0.3% Triton X-100 and 10% bovine serum albumin, followed by incubation with Alexa Fluor 488- or 647-conjugated streptavidin (1:500) for 12–24 h at 4 °C. Slices were subsequently washed in PBS (3 × 10 min), mounted on glass slides, coverslipped with Dako Fluorescent Mounting Medium, and imaged using a Leica SP8 upright confocal microscope equipped with a 20 × water-immersion objective (NA 0.7).

Electrophysiological data were analyzed using AxoGraph software. Spontaneous firing rate was calculated as the frequency of spontaneous firing events at the resting membrane potential in the absence of injected current and reported as events per minute. In NaChBac-expressing neurons, prolonged depolarization-associated firing episodes were counted as firing events for this analysis. Resting membrane potential was measured as the mean membrane voltage during a baseline period preceding current injection. Input resistance was determined from the slope of the linear relationship between steady-state voltage responses and injected current steps near the resting membrane potential. Action potential properties were quantified from the first spike evoked by the minimal depolarizing current step. Rheobase was defined as the smallest current injection required to evoke an action potential. Action potential threshold was defined as the membrane potential at which dV/dt first exceeded 50 V/s. Action potential amplitude was measured from threshold to peak, and action potential half-width was defined as the action potential duration at 50% of AP amplitude. Action potential maximum dV/dt was calculated as the peak rate of rise during the action potential upstroke. The duration of suprathreshold depolarizations (depolarizing envelope) was measured from the time the membrane voltage reached 10 mV/ms to when it returned to the same level. All electrophysiological data represents individual cells from a total of n = 4 mice (n = 2 males [n = 1 mCherry/n = 1 NaChBac]; n = 2 females [n = 1 mCherry/n = 1 NaChBac]). Surgeries for these mice were conducted at 8 weeks of age and subsequent experiments were done at 10 weeks of age.

### 4.4 Glucose Tolerance Tests (GTT) and Insulin Tolerance Tests (ITT)

GTT was conducted during week 9 and ITT during week 10, where week 1 is 2 weeks following surgery. Mice were fasted for 4 h at the start of their light cycle before mice were tail-pricked for fasting blood glucose to be measured at time 0 min using an ACCU-CHEK blood glucose monitor and strip. Mice were then intraperitoneally injected (IP) with either Glucose 2 g/kg bodyweight in physiological saline (BDH AnalaR d-Glucose Prod 10,117) or Insulin 0.75 IU/kg/lean mass in physiological saline (Novo Nordisk Actrapid® Penfill® AUST R 169,623) to specifically measure response per lean muscle mass) and then blood glucose measurements were taken at timepoint 15, 30, 60, and 120 min. Before calculating area under or above the curve, values were normalised to a percent change from the baseline blood glucose.

### 4.5 MRI, feeding cages, and behavioural tests

EchoMRI was used to assess fat and lean body mass composition during weeks 8 and 16 where week 1 is 2 weeks following surgery. Biodaq feeding cages were used to assess food intake. Ethovision XT (Noldus) was used for locomotor activity behavioural analysis and manual scoring for grooming and rearing behaviours.

*Open-field:* Mice were placed in the centre zone of an open circular arena with a diameter of 80 cm for 10 min under normal conditions and also weeks later after a 15-min restraint tube stress. During analysis on Ethovision XT (Noldus) the arena was separated into a center, middle, and outer zone.

*Sucrose-splash test:* All bedding and enrichment were removed in the home cage and then mice were subsequently sprayed on their backs with 10% sucrose solution in H_2_0 and recorded for 5 min. Since mice find sucrose pleasurable, grooming duration can be used to assess anhedonia [77].

*Saccharin preference test:* All food and drink was removed for 2 (14:00–16:00) hours over 4 days where mice were given 2 choices of either 0.1% Saccharin or H_2_0, and then the amount drank per day of each bottle was measured. The preference score was calculated by the saccharin intake (V_s_) normalized to water intake (V_w_): Preference score (%) = V_s_/(V_s_ + V_w_)⁎100.

*Exploration, anxiety, and hedonic index:* We used behavioural z-scoring as previously described [78], with all z-scores in relation to control baseline. For the exploration index we used distance moved, inner zone %, and rearing behaviour. For the anxiety index we used the inverse of the inner zone % and grooming behaviour, where the inverse of inner zone % time reflects time not spent in the inner zone. For the hedonic index we used the average preference score of the saccharin preference test % and grooming duration of the sucrose splash test. Each of these parameters correspond to behaviours in mice that are either explorative, anxious, or hedonic [79].

### 4.6 Struggling and freezing analysis

We developed custom python algorithms to analyse confidence estimation of mice in a restraint tube using DeepLabCut. The output of DeepLabCut provides 2 parameters, x,y,z coordinate data and confidence of each coordinate. Since the coordinate data are not as relevant here, as mice are constrained to a tube in space, we used confidence data to assess movement. The three features of confidence are 1) drop intensity (if the confidence values drop suddenly), 2) volatility (if the confidence values move around rapidly in a given time/frames), and 3) low confidence. This approach works because the fixed camera and position of each tube enable the algorithm to detect change in confidence (drop intensity, volatility, low confidence) if, for example, a mouse moves its paw in the tube rapidly. The code applies sensitivity or weighting to amplify bouts of higher struggle/freezing vs lower struggle/freezing by testing multiple different sensitivities in the algorithm and weighting each feature according to how well it describes the actual confidence values. For struggling this is set and adjusted for each mouse to weight if one feature better explains the struggling for that particular mouse, whilst for freezing it will test multiple sensitivities and then normalize the confidence to determine stability.

These features are scaled and filtered and a minimum bout and threshold is applied. Intensity is calculated as below:

Intensity = Σ(feature × weight) × (1 + sensitivity_adjustment) × (1 + 0.3 × intensity)

Sensitivity_adjustment is calculated as below:

Sensitivity_adjustment = (1 + (1 – sensitivity) x 0.5)

A non linear scaling is applied to (1 + 0.3 x intensity) to amplify higher intensity values.

The Struggle Persistence score is weighted so an intense and longer bout counts for more than just overall struggle.

Struggle Persistence Score = ∑(Mean Intensity × Duration) /Total Video Time x 100

The freezing algorithm works in a similar manner but tests multiple sensitivities for each video and applies a relative normalization of confidence scores.

Normalized_confidence = (confidence - minimum_video_confidence)/(maximum_video_confidence - minimum_video_confidence).

This approach measures variance of confidence to determine stability and correlates between all body parts for an adaptive threshold:

threshold = base_threshold × (1.0 / (1.0 + sensitivity × 0.1))

Hence the Freezing Burden Score accounts for the intensity of freezing over a given period of time, similar to the Struggle Persistence Score.

### 4.7 Triglycerides, non-esterified fatty acids (NEFA), glycogen, and calorific value of faeces

At the end of experimental time point, blood, muscle, and liver were collected for analysis for the chow caspase group and controls with an initial bodyweight difference. Whilst blood, muscle, liver, inguinal fat, and faeces were collected from the HFD NaChBac group and controls with a bodyweight difference. Triglycerides were determined using a FUJIFILM Wako LabAssay ™ Triglyceride kit (#291–94501) according to manufacturer's instructions for plasma and with adjusted dilution factors for tissue after extraction [80]. NEFA was determined as per manufacturer's instructions using a FUJIFILM Wako LabAssay ™ NEFA (FFA) kit (#299–94301). Tissue glycogen content was determined as per manufacturer's instructions after glucose extraction using a Sigma–Aldrich ® Glucose (GO) Assay Kit (#GAG020). A linear curve was fitted to the standards and subsequently the equation of fit was re-arranged and applied to each data point and multiplied by the dilution factor. Calorific value of faeces was determined using bomb calorimetry.

### 4.8 Corticosterone levels

Corticosterone concentrations were determined using an ELISA (Abcam #ab108821) according to manufacturer's instructions from baseline, after a 15-min restraint tube stress, and 2 h after. A 4-parametric curve was fitted to the standards and subsequently the equation of fit was re-arranged and applied to each data point and multiplied by the dilution factor.

### 4.9 Immunohistochemistry

To confirm viral expression, animals were deeply anesthetized with isoflurane and perfused with 0.05 M PBS, followed by 4% paraformaldehyde. Brains were postfixed in 4% paraformaldehyde overnight at 4 °C, then placed in 30% sucrose. Brains were cut at 40 μm on a cryostat, and every fourth section was collected and stored in cryoprotectant at −20 °C. For staining, sections were washed in 0.1 M PBS (3 × 10 min) and then blocked for 60 min in 4% normal horse serum in PBS + 0.3% Triton-X. Sections were incubated in primary antibody (chicken anti-GFP; ab13970; abcam) diluted 1:1000 in blocking solution overnight at 4 °C. The next day, sections were washed in 0.1 M PB (3 × 10 min) and incubated with secondary antibody (goat anti-chicken; AB_2,337,390; Jackson ImmunoResearch) at a dilution of 1:500 in 0.1 M PB for 2 h at room temperature. Following a final wash, sections were mounted onto SuperFrost slides (Thermo Fisher Scientific) with VECTASHIELD antifade mounting medium with DAPI (Vector Labs). Post-curing, slides were then sealed with nail polish and stored at 4 °C. Sections were imaged under an upright fluorescence microscope (Zeiss Axio Imager 2; Zeiss, Germany). Tile acquisition for appropriate channels was conducted whereby exposure, gain, and offset were adjusted at either site of highest expression or site of interest. All animals were histologically validated for caspase ablation or NaChBac expression.

### 4.10 Translating ribosomal affinity purification (TRAP) assay and RNA sequencing

TRAP was performed based on a combination of previously used protocols [81], [82], [83]. Followed by RNA extraction using QIAzol lysis reagent (Qiagen) and Monarch® Spin RNA Cleanup Kit (10 μg; New England Biolabs). 11 samples were initially ran on QC, but one was excluded due to low RNA concentration. Samples were enriched for small RNA fragments possessing 5′ monophosphate and 3’ hydroxyl termini and were processed for library construction. RNA integrity was determined via capillary electrophoresis (Fragment Analyzer, Agilent). Low RIN samples were not excluded as TRAP-seq yields fragmented and non-intact RNA from bead-based immunoprecipitation rather than degradation of RNA [81], [84]. Library preparation was conducted using the NEBNext Low-Bias Small RNA Library Prep Kit (New England Biolabs), to capture fragmented mRNA and small RNA and sequenced on an Illumina NovaSeq X Plus (Illumina) with XLEAP-SBS chemistry with paired-end 150 b reads. With approximately 10 M reads per sample, raw reads were compressed to FASTQ files.

FASTQ files were processed, analysed, and graphed using custom Python scripts available at: https://github.com/VasiliDrakopoulos/RNAseq-Analysis. Files are assessed by FASTQC (v0.11.9) and then aggregated across samples using MultiQC (v1.13). Adapter sequences specific to the NEBNext Low-Bias Small RNA Library Prep Kit (New England Biolabs) were removed and low-quality bases were trimmed using Cutadapt (v4.4; reads shorter than 15 nucleotides after trimming were excluded). The trimmed reads were aligned to the mouse genome (GRCm38) using HISAT (v2.2.1). These alignments were sorted and indexed using SAMtools (v1.16). Gene quantification was conducted using featureCounts (v2.0.3) and then parsed using pandas. Any gene with zero cumulative reads across samples was excluded. The final gene counts are then exported as a csv file. Differential expression analysis was conducted using PyDESeq2 [85]. Matplotlib was used to create graphs and Microsoft PowerPoint for network visualization. MicroRNA targets were found using https://www.targetscan.org/vert_80/ [86]. Pathway enrichment analysis on microRNAs was conducted using DIANA-miRPath v4.0 [87]. Pathway enrichment using KEGG for protein coding mRNAs was conducted using ShinyGO 0.85.1 [88]. For all RNA related information, see RNA_data.

## 5 Statistics

All statistical analysis was conducted on GraphPad Prism for PC or custom scripts for RNA analysis. All data is represented as mean ± SEM with p < 0.05⁎ considered statistically significant unless otherwise stated in figure legends. All data was tested for normality using the Shapiro–Wilk Test, if deemed non-parametric, a Mann–Whitney test was conducted over a t-test. ROUT outlier tests were conducted on the struggling and freezing algorithm with a Q = 0.1%. Exact statistical tests and *n* values can be found in figures and legends.

## CRediT authorship contribution statement

**Vasilios Drakopoulos:** Writing – review & editing, Writing – original draft, Visualization, Validation, Software, Methodology, Investigation, Formal analysis, Data curation, Conceptualization. **Xiaolong Feng:** Writing – review & editing, Methodology, Investigation, Formal analysis, Data curation. **Daria J. Paulis:** Visualization, Investigation. **Abigail C. Wust:** Investigation. **Lotus S. Jeffs:** Investigation. **Romana Stark:** Methodology. **Greg J. Stuart:** Writing – review & editing, Resources, Methodology. **Claire J. Foldi:** Writing – review & editing, Supervision. **Alex Reichenbach:** Writing – review & editing, Supervision, Methodology, Investigation, Conceptualization. **Zane B. Andrews:** Writing – review & editing, Visualization, Supervision, Resources, Project administration, Methodology, Funding acquisition, Data curation, Conceptualization.

## Ethics declaration

This study was conducted in accordance with the ARRIVE (Animal Research: Reporting of In Vivo Experiments) guidelines. This study was approved by the MUEC1.

## Funding

V.D. acknowledges support from an Australian Government Research Training Program (RTP) Scholarship. This work was supported via grants appointed to Z.B.A. from the National Health and Medical Research Council of Australia (APP2013337, APP1154974).

## Declaration of competing interest

All other authors report no conflict of interest and have nothing to disclose.

## Data Availability

Data will be made available on request.

## Appendix A Supplementary data

Multimedia component 1 Body weight gain, body composition gain, GTT, and ITT of chow and HFD caspase males and females. (A) Body weight gain in grams of control and caspase chow fed males. (B) Body weight gain in grams of control and caspase chow fed females. (C) Fat mass gain in grams of control and caspase chow fed males. (D) Fat mass gain in grams of control and caspase chow fed females. (E) Lean mass gain in grams of control and caspase chow fed males. (F) Lean mass gain in grams of control and caspase chow fed females. (G) Glucose tolerance test (GTT) area under the curve (AUC) for chow males. (H) Insulin tolerance test (ITT) area above curve for chow males. (I) GTT AUC for chow females. (J) ITT AAC for chow females. (K) Body weight gain in grams of control and caspase HFD fed males. (L) Body weight gain in grams of control and caspase HFD fed females. (M) Fat mass gain in grams of control and caspase HFD fed males. (N) Fat mass gain in grams of control and caspase HFD fed females. (O) Lean mass gain in grams of control and caspase HFD fed males. (P) Lean mass gain in grams of control and caspase HFD fed females. (Q) GTT AUC for HFD males. (R) ITT AAC for HFD males. (S) GTT AUC for HFD females. (T) ITT AAC for HFD females. Two-way repeated measures ANOVA with Šídák's multiple comparisons test (A, B, C, D, E, F, K, L, M, N, O, P). For males (A, E, M, O) n = 9 for caspase, (C, K) n = 8 for caspase, and (A, B, C, D, E, F) n = 6 for controls, (K, M, O) n = 9 for controls. For females (B, D, F) n = 10 for caspase, (L, N, F) n = 9 for caspase and n = 6 for controls. Unpaired t test (G, H, I, J, Q, R, S) and Welch’s t-test (T). For males and females on chow, and females on HFD (G, H, I, J, S, T) n = 6 for controls and n = 9 for caspase. For males on chow (E, F) n = 9 for both controls and caspase. Data is represented as mean ± SEM, ∗∗p < 0.01.

Multimedia component 2 Feeding cage data of chow fed caspase males and females. (A) Cumulative food intake of males. (B) Average food intake of the light and dark cycle for males. (C) 3 hours of fasted re-feeding food intake of males. (D) Cumulative food intake of females. (E) Average food intake of the light and dark cycle for females. (F) 3 hours of fasted re-feeding food intake of females. (G) Average hourly number of bouts for males. (H) Average number of bouts of the light and dark cycle for males. (I) Average bout duration of the light and dark cycle for males. (J) Average hourly number of bouts for females. (K) Average number of bouts of the light and dark cycle for females. (L) Average bout duration of the light and dark cycle for females. Two-way repeated measures ANOVA with Šídák's multiple comparisons test (A, C, D, F), with Uncorrected Fisher’s LSD (B, E, H, I, K, L), or Tukey’s multiple comparisons test (G, J). For males (A, B, C, G, H, I) n = 5 for controls and n = 8 for caspase. For females (D, E, F, J, K, L) n = 6 for controls and n = 10 for caspase. Data is represented as mean ± SEM, ∗∗p < 0.01, ∗∗∗p < 0.001, ∗∗∗∗p < 0.0001.

Multimedia component 3 Electrophysiology of control and NaChBac mice. (A) Average action potential waveform centred on the peak for control (n = 7) and NaChBac (n = 10) neurons. Solid lines represent the mean and shaded areas represent SEM. (B) Phase plot of Action potential for control (n = 7) and NaChBac (n = 7) neurons. Solid lines represent the mean and shaded areas represent SEM. Comparison on electrophysiological properties in control (grey) and NaChBac (purple) mPFC neurons projecting to the LH: (C) Resting membrane potential, (D) AP rheobase, (E) Action potential (AP) threshold, (F) AP half width, and (G) AP amplitude. There was no statistical difference between control and mPFC-LHNaChBac neurons for any of these electrophysiological properties. Unpaired t-test (C, D, F), Welch’s t-test (E), and Mann-Whitney test (G). For controls (A, C, D, E, F, G) n = 7 and for NaChBac n = 10, (G) n = 6. Data is represented as mean ± SEM.

Multimedia component 4 Electrophysiology between NaChBac sexes. (A) Representative images showing EGFP expression in male NaChBac mice in the mPFC neurons (green) and a biocytin-filled recorded neuron (red). (B) Representative whole-cell current-clamp recording showing spontaneous firing of a mPFC-LHNaChBac neuron at resting membrane potential in a male mouse. (C) Representative whole-cell current-clamp recording from a mPFC-LHNaChBac neuron during 1 second hyperpolarising and depolarising current steps in a male mouse. (D) Average action potential waveform centred on the peak for mPFC-LHNaChBac neurons in female (n = 6) and male (n = 7). Solid lines represent the mean and shaded areas represent SEM. (E) Resting membrane potential of mPFC-LHNaChBac neurons in male and female. (F) Input resistance of mPFC-LHNaChBac neurons in male and female. (G) Spontaneous firing rates of mPFC-LHNaChBac neurons in male and females. (H) Maximum rise dV/dt of mPFC-LHNaChBac neurons in male and female. (I) Rheobase of mPFC-LHNaChBac neurons in male and female. (J) Action potential threshold of mPFC-LHNaChBac neurons in male and female. (K) Action potential amplitude from threshold of mPFC-LHNaChBac neurons in male and female. (L) Action potential half-width of mPFC-LHNaChBac neurons in male and female. (M) Depolarization duration of mPFC-LHNaChBac neurons in male and female. Unpaired t-test (E, F, G, H, I, J, K, L, M). For males (E) n = 7, (F, G, H, L) n = 6, (I, K, M) n = 4 , (J) n = 5. For females (E) n = 7, (F, G, H, I, K, L, M) n = 6, (J) n = 5. All electrophysiological data is from individual cells from n = 1 from each group where mice were 10 weeks of age conducted 2 weeks after surgery. Data is represented as mean ± SEM.

Multimedia component 5 Body weight gain, body composition gain, GTT, and ITT of chow and HFD NaChBac males and females. (A) Body weight gain in grams of control and NaChBac chow fed males. (B) Body weight gain in grams of control and NaChBac chow fed females. (C) Fat mass gain in grams of control and NaChBac chow fed males. (D) Fat mass gain in grams of control and NaChBac chow fed females. (E) Lean mass gain in grams of control and NaChBac chow fed males. (F) Lean mass gain in grams of control and NaChBac chow fed females. (G) Glucose tolerance test (GTT) area under the curve (AUC) for chow males. (H) Insulin tolerance test (ITT) area above curve for chow males. (I) GTT AUC for chow females. (J) ITT AAC for chow females. (K) Body weight gain in grams of control and NaChBac HFD fed males. (L) Body weight gain in grams of control and NaChBac HFD fed females. (M) Fat mass gain in grams of control and NaChBac HFD fed males. (N) Fat mass gain in grams of control and NaChBac HFD fed females. (O) Lean mass gain in grams of control and NaChBac HFD fed males. (P) Lean mass gain in grams of control and NaChBac HFD fed females. (Q) GTT AUC for HFD males. (R) ITT AAC for HFD males. (S) GTT AUC for HFD females. (T) ITT AAC for HFD females. Two-way repeated measured ANOVA with Šídák's multiple comparisons test (A, B, C, D, E, F, K, L, M, N, O, P). For males (A, C, E, O) n = 10 for controls (K, M, O) n = 9 for controls, and n = 9 for NaChBac. For females (B, D, F, L, N, P) n = 10 for controls, and n = 9 for NaChBac, (L, N, P) n = 10 for NaChBac. Unpaired t-test (G, K, Q, R, S), Welch’s t-test (T), and Mann-Whitney test (H, I). For males and females on chow (G, H, I, J), n = 10 for controls and n = 9 for NaChBac. For males on HFD (Q, R) n = 9 for both controls and NaChBac. For females on HFD (S, T) n = 10 for both controls and NaChBac. Data is represented as mean ± SEM.

Multimedia component 6 Open-field, struggling, and freezing of chow fed caspase males and females. (A) Open-field distance moved males. (B) Latency to outer wall of open-field males. (C) Open-field grooming behaviour males. (D) Open-field rearing behaviour males. (E) Open-field inner zone % males. (F) Open-field middle zone % males. (G) Open-field outer zone % males. (H) Open-field distance moved females. (I) Latency to outer wall of open-field females. (J) Open-field grooming behaviour females. (K) Open-field rearing behaviour females. (L) Open-field inner zone % females. (M) Open-field middle zone % females. (N) Open-field outer zone % females. (O) Struggling % in 15-min restraint stress tube. (P) Struggle bouts in 15-min restraint stress tube. (Q) Max struggle bout duration in 15-min restraint stress tube. (R) Mean struggle intensity in 15-min restraint stress tube. (S) Max struggle intensity in 15-min restraint stress tube. (T) Freezing % in 15-min restraint stress tube. (U) Freezing bouts in 15-min restraint stress tube. (V) Max freezing bout duration in 15-min restraint stress tube. (W) Mean freezing intensity in 15-min restraint stress tube. (X) Max freezing intensity in 15-min restraint stress tube. Two-way repeated measures ANOVA with Uncorrected Fisher’s LSD (A, B, C, D, E, F, G, H, I, J, K, L, M, N) and ordinary two-way ANOVA (O, P, Q, R, S, T, U, V, W, X). For open-field, struggling, and freezing data for caspase males and females, (A, B, C, D, E, F, G, H, I, J, K, L, M, N, O, P, R, S, T, U, V, W, X) n = 6 for controls, n = 9 for caspase males (T) , n = 10 for caspase females (T) , (Q) n = 4 for control males, (T) n = 4 for control males and females, (Q) n = 8 for caspase males, (Q) n = 9 for caspase females. Data is represented as mean ± SEM, ∗p < 0.05, ∗∗p &lt; 0.01, ∗∗∗p < 0.001.

Multimedia component 7 Open-field, struggling, and freezing of HFD fed caspase males and females. (A) Open-field distance moved males. (B) Latency to outer wall of open-field males. (C) Open-field grooming behaviour males. (D) Open-field rearing behaviour males. (E) Open-field inner zone % males. (F) Open-field middle zone % males. (G) Open-field outer zone % males. (H) Open-field distance moved females. (I) Latency to outer wall of open-field females. (J) Open-field grooming behaviour females. (K) Open-field rearing behaviour females. (L) Open-field inner zone % females. (M) Open-field middle zone % females. (N) Open-field outer zone % females. (O) Struggling % in 15-min restraint stress tube. (P) Struggle bouts in 15-min restraint stress tube. (Q) Max struggle bout duration in 15-min restraint stress tube. (R) Mean struggle intensity in 15-min restraint stress tube. (S) Max struggle intensity in 15-min restraint stress tube. (T) Freezing % in 15-min restraint stress tube. (U) Freezing bouts in 15-min restraint stress tube. (V) Max freezing bout duration in 15-min restraint stress tube. (W) Mean freezing intensity in 15-min restraint stress tube. (X) Max freezing intensity in 15-min restraint stress tube. Two-way repeated measures ANOVA with Uncorrected Fisher’s LSD (A, B, C, D, E, F, G, H, I, J, K, L, M, N) and ordinary two-way ANOVA (O, P, Q, R, S, T, U, V, W, X). For open-field, struggling, and freezing data for caspase males, (A, B, C, D, E, F, G, O, P, R, S, T, U, V, W, X) n = 9 for controls, n = 9 for caspase, (Q) n = 8 for controls and caspase. For caspase females (H, I, J, K, L, M, N, O, P, Q, R, S, T, U, V, W, X) n = 6 for controls and n = 9 for caspase. Data is represented as mean ± SEM, ∗p < 0.05, ∗∗p < 0.01.

Multimedia component 8 Saccharin preference test and sucrose splash test of chow and HFD fed caspase males and females. (A) Saccharin preference % of chow fed caspase males. (B) Total amount drank of water, or 0.1% saccharin of chow fed caspase males. (C) Grooming duration during sucrose splash test of chow fed caspase males. (D) Saccharin preference % of chow fed caspase females. (E) Total amount drank of water, or 0.1% saccharin of chow fed caspase females. (F) Grooming duration during sucrose splash test of chow fed caspase females. (G) Saccharin preference % of HFD fed caspase males. (H) Total amount drank of water, or 0.1% saccharin of HFD fed caspase males. (I) Grooming duration during sucrose splash test of HFD fed caspase males. (J) Saccharin preference % of HFD fed caspase females. (K) Total amount drank of water, or 0.1% saccharin of HFD fed caspase females. (L) Grooming duration during sucrose splash test of HFD fed caspase females. Two-way repeated measures ANOVA with Šídák's multiple comparisons test (A, D) or with Uncorrected Fisher’s LSD (B, E), Unpaired t-test (C, F) and mixed-effects analysis with Šídák's multiple comparisons test (G, J) or with Uncorrected Fisher’s LSD (H), Mann-Whitney test (I). For males on chow (A, B, C) n = 5 for controls and n = 8 for caspase. For females on chow (D, E, F) n = 6 for controls and n = 10 for caspase. For males on HFD (G) day 1 for controls and caspase n = 9, day 2, 3, 4 for controls and day 2 and 4 for caspase n = 8, day 3 for caspase n = 6. (H) water and 0.1% saccharin controls and 0.1% saccharin caspase n = 7, water caspase n = 8. (I) n = 8 for controls and caspase. For females on HFD (J) n = 6 for day 1 controls, day 2, 3, and 4 n = 5 controls, for caspase day 1 n = 8, for day 2, 3, and 4 n = 9. (K) water and 0.1% saccharin controls n = 5, water caspase n = 9, and 0.1% saccharin caspase n = 8. (L) n = 6 for controls and n = 9 for caspase. Data is represented as mean ± SEM, ∗∗p < 0.01, ∗∗∗p < 0.001.

Multimedia component 9 Open-field, struggling, and freezing of chow fed NaChBac males and females. (A) Open-field distance moved males. (B) Latency to outer wall of open-field males. (C) Open-field grooming behaviour males. (D) Open-field rearing behaviour males. (E) Open-field inner zone % males. (F) Open-field middle zone % males. (G) Open-field outer zone % males. (H) Open-field distance moved females. (I) Latency to outer wall of open-field females. (J) Open-field grooming behaviour females. (K) Open-field rearing behaviour females. (L) Open-field inner zone % females. (M) Open-field middle zone % females. (N) Open-field outer zone % females. (O) Struggling % in 15-min restraint stress tube. (P) Struggle bouts in 15-min restraint stress tube. (Q) Max struggle bout duration in 15-min restraint stress tube. (R) Mean struggle intensity in 15-min restraint stress tube. (S) Max struggle intensity in 15-min restraint stress tube. (T) Freezing % in 15-min restraint stress tube. (U) Freezing bouts in 15-min restraint stress tube. (V) Max freezing bout duration in 15-min restraint stress tube. (W) Mean freezing intensity in 15-min restraint stress tube. (X) Max freezing intensity in 15-min restraint stress tube. Mixed-effects analysis with Uncorrected Fisher’s LSD (A, B, C, E, F, G, H, I, J, K, L, M, N) and ordinary two-way ANOVA (O, P, Q, R, S, T, U, V, W, X). For open-field for NaChBac males non-stressed condition (A, B, C, D, E, F, G) n = 8 for controls and n = 6 for NaChBac, (A, B, C, D, E, F, G, H, I, J, K, L, M, N) in the stressed condition for males and females n = 10 for controls and n = 9 for NaChBac. For open-field data for NaChBac females (H, I, J, K, L, M, N) n = 9 for controls and n = 6 for NaChBac. For struggling for males (O, Q, R, S) n = 10 for controls, (P) n = 9 for controls and (O, Q, R) n = 8 for NaChBac, (P, S) n = 9 for NaChBac, (Q) n = 7 for NaChBac, for females (O, P, Q, R, S) n = 9 for controls and NaChBac each, (Q) n = 7 for NaChBac. For freezing for males (T, V, W, X) n = 6 for controls, (U) n = 5 for controls, and (T, U, V, W, X) n = 7 for NaChBac, and (T, U, V, W, X) n = 8 for controls and NaChBac females. Data is represented as mean ± SEM, ∗p < 0.05, ∗∗p < 0.01.

Multimedia component 10 Open-field, struggling, and freezing of HFD fed NaChBac males and females. (A) Open-field distance moved males. (B) Latency to outer wall of open-field males. (C) Open-field grooming behaviour males. (D) Open-field rearing behaviour males. (E) Open-field inner zone % males. (F) Open-field middle zone % males. (G) Open-field outer zone % males. (H) Open-field distance moved females. (I) Latency to outer wall of open-field females. (J) Open-field grooming behaviour females. (K) Open-field rearing behaviour females. (L) Open-field inner zone % females. (M) Open-field middle zone % females. (N) Open-field outer zone % females. (O) Struggling % in 15-min restraint stress tube. (P) Struggle bouts in 15-min restraint stress tube. (Q) Max struggle bout duration in 15-min restraint stress tube. (R) Mean struggle intensity in 15-min restraint stress tube. (S) Max struggle intensity in 15-min restraint stress tube. (T) Freezing % in 15-min restraint stress tube. (U) Freezing bouts in 15-min restraint stress tube. (V) Max freezing bout duration in 15-min restraint stress tube. (W) Mean freezing intensity in 15-min restraint stress tube. (X) Max freezing intensity in 15-min restraint stress tube. Mixed-effects analysis with Uncorrected Fisher’s LSD (A, B, C, E, F, G, H, I, J, K, L, M, N) and ordinary two-way ANOVA (O, P, Q, R, S, T, U, V, W, X). For open-field for NaChBac males non-stressed condition (A, B, C, D, E, F, G) n = 9 for controls and n = 7 for NaChBac, (A, B, C, D, E, F, G, H, I, J, K, L, M, N) in the stressed condition for males and females n = 9 for controls and n = 8 for NaChBac. For open-field data for NaChBac females (H, I, J, K, L, M, N) n = 10 for controls and for non-stressed condition (H, I, J, K, L, M, N) n = 8 for NaChBac, for stressed condition n = 9 for NaChBac. Struggling for males (O, P, Q, R, S) n = 9 for controls and (O, P, R, S) n = 9 for NaChBac, (Q) n = 8 for NaChBac, for females (O) n = 9 for controls and (Q) NaChBac, (P, R, S) n = 10 for controls and NaChBac, and (Q) n = 7 for controls. Freezing for males (T, U, V, W, X) n = 9 for controls and NaChBac each, for females (T, U, V, W, X) n = 10 for controls and (T, U, V, W, X) n = 9 for NaChBac. Data is represented as mean ± SEM, ∗p < 0.05, ∗∗p < 0.01.

Multimedia component 11 Saccharin preference test and sucrose splash test of chow and HFD fed NaChBac males and females. (A) Saccharin preference % of chow fed NaChBac males. (B) Total amount drank of water, or 0.1% saccharin of chow fed NaChBac males. (C) Grooming duration during sucrose splash test of chow fed NaChBac males. (D) Saccharin preference % of chow fed NaChBac females. (E) Total amount drank of water, or 0.1% saccharin of chow fed NaChBac females. (F) Grooming duration during sucrose splash test of chow fed NaChBac females. (G) Saccharin preference % of HFD fed NaChBac males. (H) Total amount drank of water, or 0.1% saccharin of HFD fed NaChBac males. (I) Grooming duration during sucrose splash test of HFD fed NaChBac males. (J) Saccharin preference % of HFD fed NaChBac females. (K) Total amount drank of water, or 0.1% saccharin of HFD fed NaChBac females. (L) Grooming duration during sucrose splash test of HFD fed NaChBac females. Mixed-effects analysis with Šídák's multiple comparisons test (A, B, D, E, G) or with Uncorrected Fisher’s LSD (H), Mann-Whitney test (C, F), Unpaired t-test (I, L), and two-way repeated measures ANOVA with Šídák's multiple comparisons test (J) or with Uncorrected Fisher’s LSD (K). For males on chow (A) day 1, 2, and 3 n = 10 for controls, day 4 n = 9 for controls and n = 9 for NaChBac. (B) water controls and NaChBac n = 9, 0.1% saccharin controls n = 10 for controls and n = 8 for NaChBac. (C) n= 10 for controls and n = 9 for NaChBac. For females on chow (D) n = 10 for controls and n = 9 for NaChBac. For males on HFD (G, H, I) n = 9 for controls and NaChBac, except n = 8 for controls on day 4 and 0.1 % saccharin total amount drank and grooming duration of sucrose splash test. For females on HFD (J, K) n = 10 for controls and NaChBac, except n = 9 for controls for grooming duration of sucrose splash test. Data is represented as mean ± SEM, ∗∗p < 0.01, ∗∗p < 0.01.
