# Supplementary material for: A cortico-hypothalamic circuit regulates metabolism and stress resilience in a sex-specific manner
Source: Mol Metab. 2026 Oct 22;114:102459. doi: 10.1016/j.molmet.2026.102459 (PMC13755591; doi:10.1016/j.molmet.2026.102459)
Supplement: Supplementary file 1 [file mmc1.pdf]

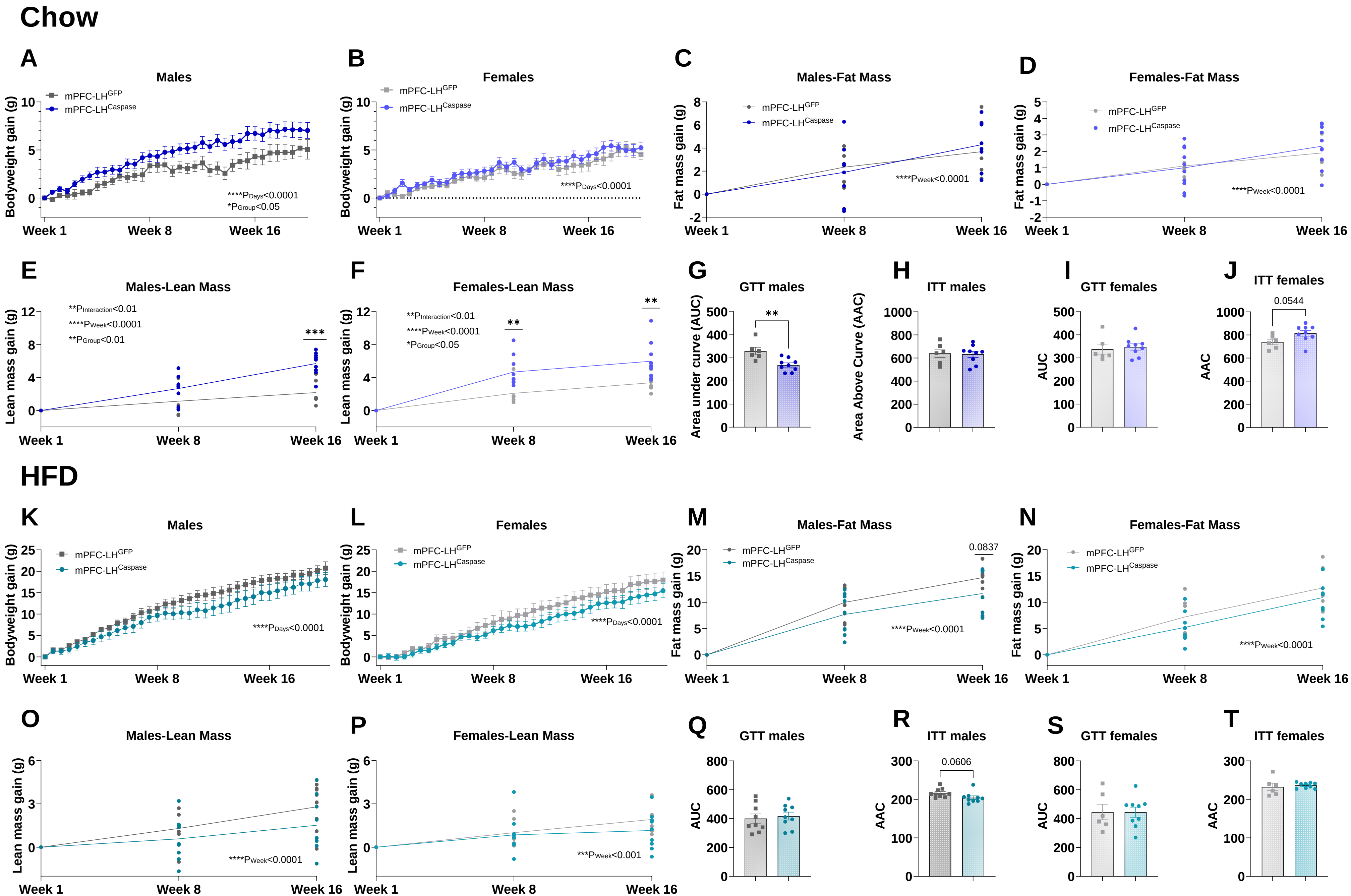

Chow
A
Males
mPFC-LH^GFP
mPFC-LH^Caspase
Bodyweight gain (g)
****P_Days<0.0001
*P_Group<0.05
Week 1
Week 8
Week 16
B
Females
****P_Days<0.0001
C
Males-Fat Mass
Fat mass gain (g)
****P_Week<0.0001
D
Females-Fat Mass
E
Males-Lean Mass
Lean mass gain (g)
**P_Interaction<0.01
****P_Week<0.0001
**P_Group<0.01
F
Females-Lean Mass
*P_Group<0.05
G
GTT males
Area under curve (AUC)
H
ITT males
Area Above Curve (AAC)
I
GTT females
AUC
J
ITT females
AAC
0.0544
HFD
K
Males
L
Females
M
Males-Fat Mass
0.0837
N
Females-Fat Mass
O
Males-Lean Mass
P
Females-Lean Mass
***P_Week<0.001
Q
GTT males
R
ITT males
0.0606
S
GTT females
T
ITT females
