# Supplementary material for: A cortico-hypothalamic circuit regulates metabolism and stress resilience in a sex-specific manner
Source: Mol Metab. 2026 Oct 22;114:102459. doi: 10.1016/j.molmet.2026.102459 (PMC13755591; doi:10.1016/j.molmet.2026.102459)
Supplement: Supplementary file 2 [file mmc10.pdf]

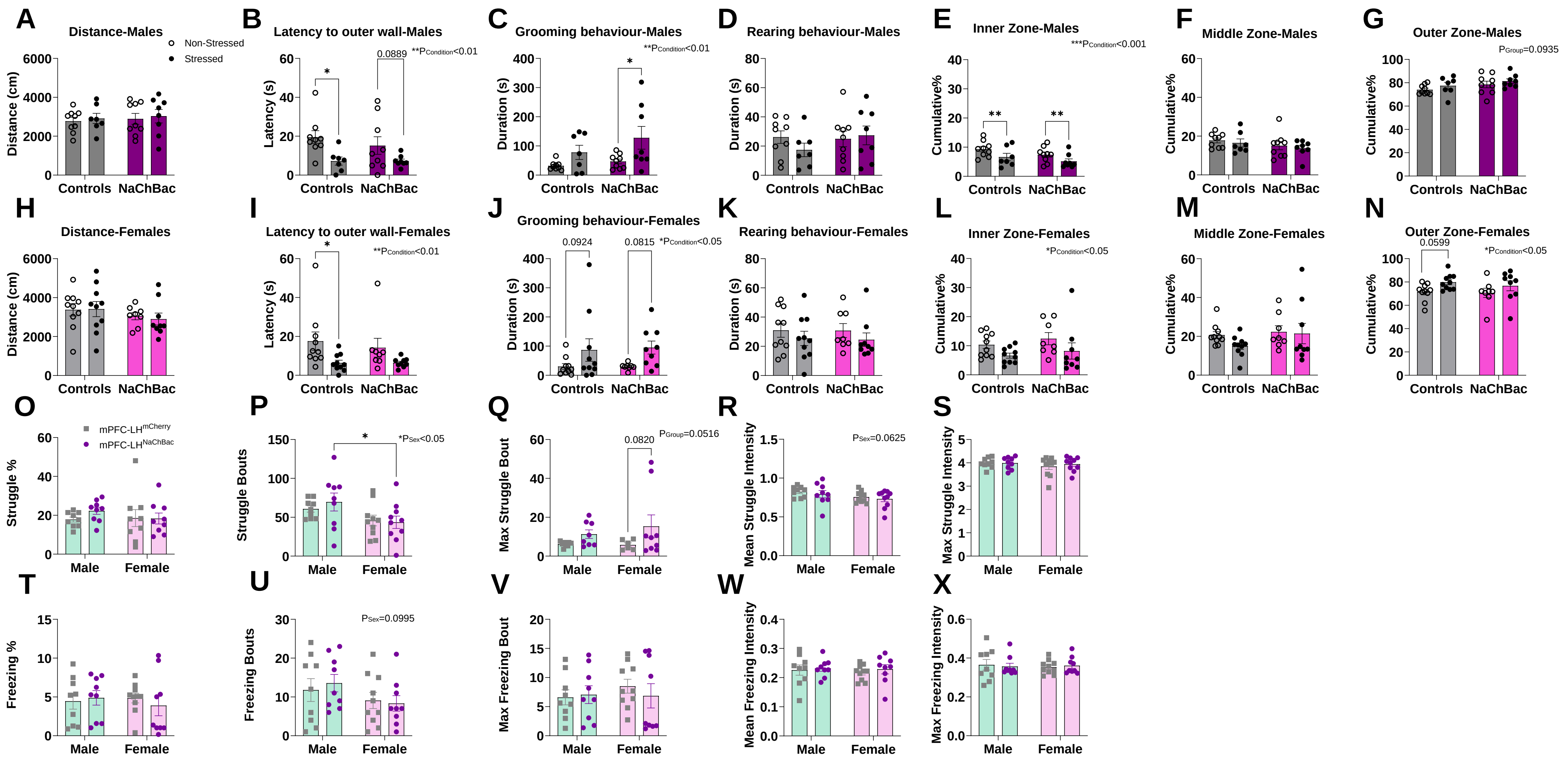
A
Distance-Males
Non-Stressed
Stressed
Distance (cm)
Controls
NaChBac
B
Latency to outer wall-Males
0.0889
**PCondition<0.01
Latency (s)
C
Grooming behaviour-Males
**PCondition<0.01
Duration (s)
D
Rearing behaviour-Males
Duration (s)
E
Inner Zone-Males
***PCondition<0.001
Cumulative%
F
Middle Zone-Males
Cumulative%
G
Outer Zone-Males
PGroup=0.0935
Cumulative%
H
Distance-Females
Distance (cm)
I
Latency to outer wall-Females
**PCondition<0.01
Latency (s)
J
Grooming behaviour-Females
0.0924
0.0815
*PCondition<0.05
Duration (s)
K
Rearing behaviour-Females
Duration (s)
L
Inner Zone-Females
*PCondition<0.05
Cumulative%
M
Middle Zone-Females
Cumulative%
N
Outer Zone-Females
0.0599
*PCondition<0.05
Cumulative%
O
mPFC-LHmCherry
mPFC-LHNaChBac
Struggle %
Male
Female
P
*PSex<0.05
Struggle Bouts
Q
0.0820
PGroup=0.0516
Max Struggle Bout
R
PSex=0.0625
Mean Struggle Intensity
S
Max Struggle Intensity
T
Freezing %
U
PSex=0.0995
Freezing Bouts
V
Max Freezing Bout
W
Mean Freezing Intensity
X
Max Freezing Intensity
