# Supplementary material for: A cortico-hypothalamic circuit regulates metabolism and stress resilience in a sex-specific manner
Source: Mol Metab. 2026 Oct 22;114:102459. doi: 10.1016/j.molmet.2026.102459 (PMC13755591; doi:10.1016/j.molmet.2026.102459)
Supplement: Supplementary file 3 [file mmc11.pdf]

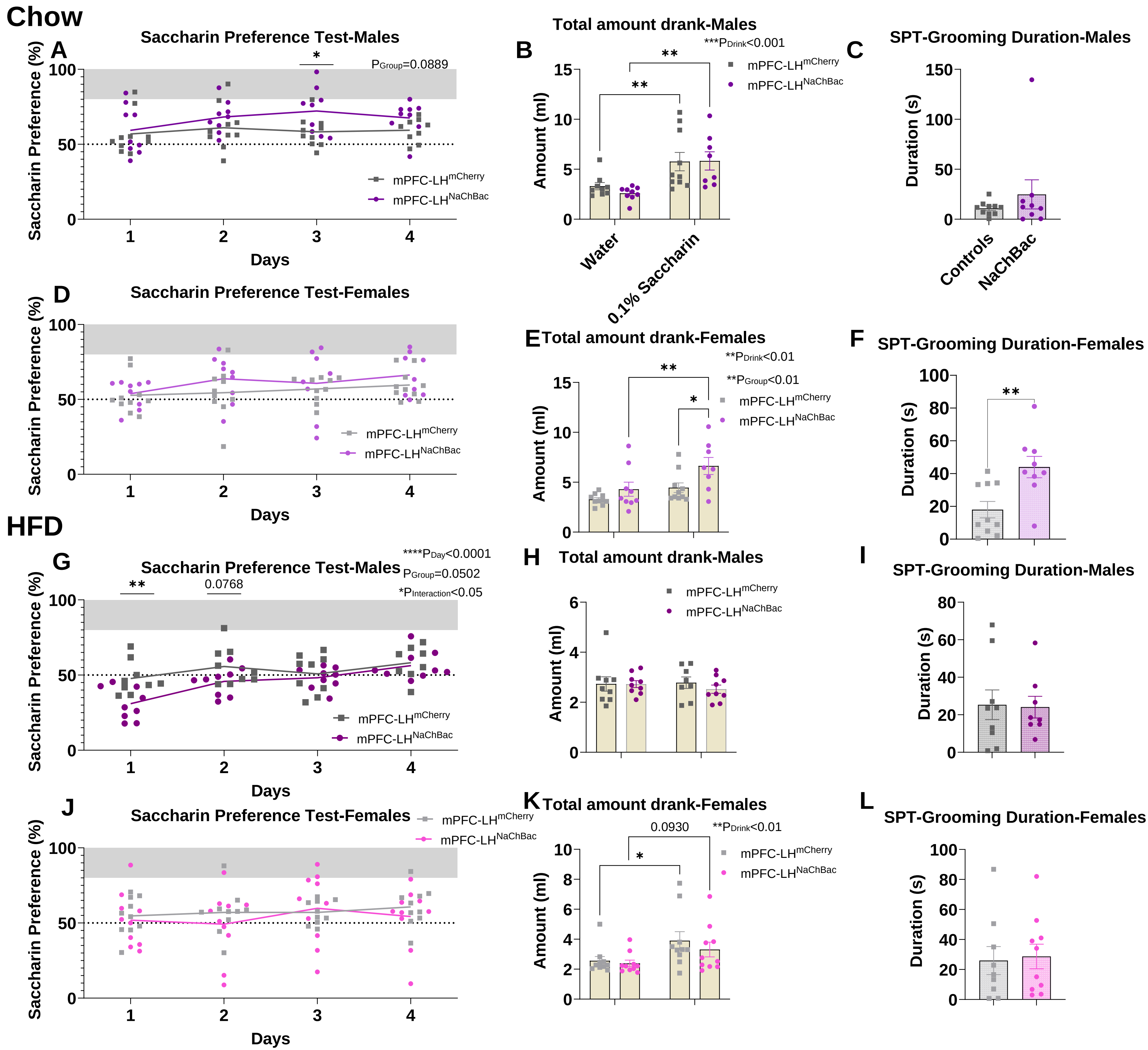

Chow
A
Saccharin Preference Test-Males
P_{Group}=0.0889
*
Saccharin Preference (%)
Days
mPFC-LH^{mCherry}
mPFC-LH^{NaChBac}
B
Total amount drank-Males
***P_{Drink}<0.001
**
**
Amount (ml)
Water
0.1% Saccharin
C
SPT-Grooming Duration-Males
Duration (s)
Controls
NaChBac
D
Saccharin Preference Test-Females
E
Total amount drank-Females
**P_{Drink}<0.01
**P_{Group}<0.01
*
F
SPT-Grooming Duration-Females
HFD
G
Saccharin Preference Test-Males
****P_{Day}<0.0001
P_{Group}=0.0502
*$P_{Interaction}$<0.05
0.0768
H
Total amount drank-Males
I
SPT-Grooming Duration-Males
J
Saccharin Preference Test-Females
K
Total amount drank-Females
0.0930
**P_{Drink}<0.01
L
SPT-Grooming Duration-Females
