# Supplementary material for: A cortico-hypothalamic circuit regulates metabolism and stress resilience in a sex-specific manner
Source: Mol Metab. 2026 Oct 22;114:102459. doi: 10.1016/j.molmet.2026.102459 (PMC13755591; doi:10.1016/j.molmet.2026.102459)
Supplement: Supplementary file 4 [file mmc2.pdf]

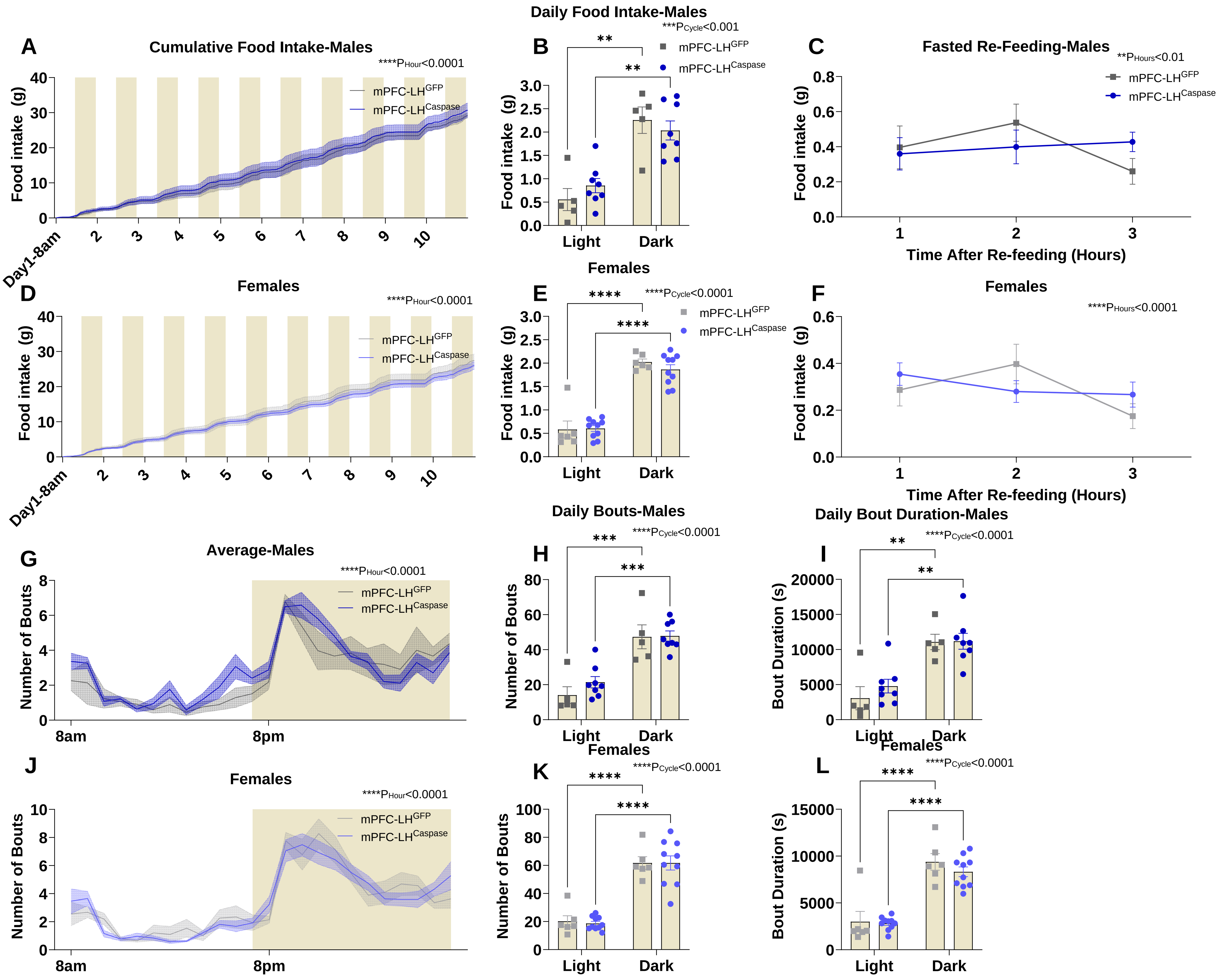

Daily Food Intake-Males
A
Cumulative Food Intake-Males
****PHour<0.0001
mPFC-LHGFP
mPFC-LHCaspase
Food intake (g)
Day1-8am
B
***PCycle<0.001
Light
Dark
C
Fasted Re-Feeding-Males
**PHours<0.01
Time After Re-feeding (Hours)
Females
D
E
****PCycle<0.0001
F
****PHours<0.0001
Daily Bouts-Males
Daily Bout Duration-Males
G
Average-Males
Number of Bouts
8am
8pm
H
I
Bout Duration (s)
J
K
L
