# Supplementary material for: A cortico-hypothalamic circuit regulates metabolism and stress resilience in a sex-specific manner
Source: Mol Metab. 2026 Oct 22;114:102459. doi: 10.1016/j.molmet.2026.102459 (PMC13755591; doi:10.1016/j.molmet.2026.102459)
Supplement: Supplementary file 5 [file mmc3.pdf]

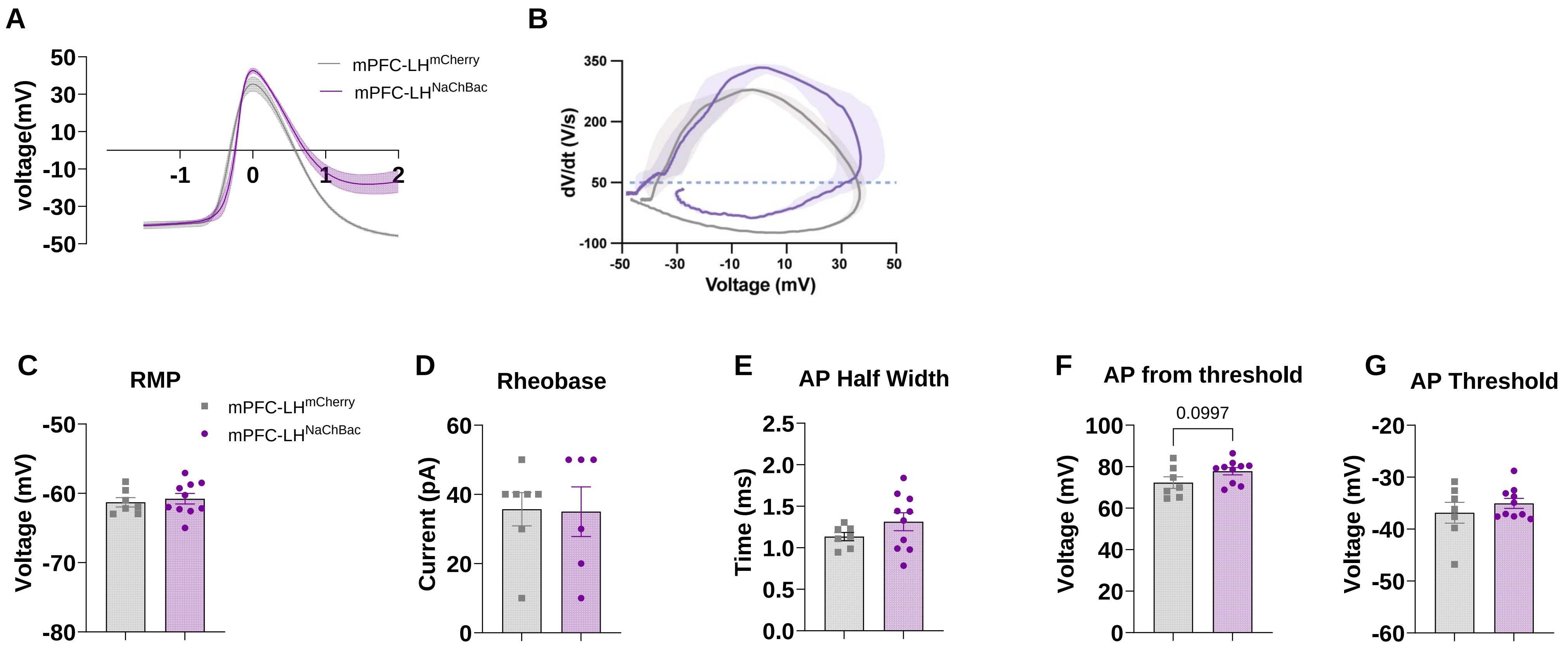

A
voltage(mV)
mPFC-LH^mCherry
mPFC-LH^NaChBac
B
dV/dt (V/s)
Voltage (mV)
C
RMP
Voltage (mV)
D
Rheobase
Current (pA)
E
AP Half Width
Time (ms)
F
AP from threshold
0.0997
Voltage (mV)
G
AP Threshold
Voltage (mV)
