# Supplementary material for: A cortico-hypothalamic circuit regulates metabolism and stress resilience in a sex-specific manner
Source: Mol Metab. 2026 Oct 22;114:102459. doi: 10.1016/j.molmet.2026.102459 (PMC13755591; doi:10.1016/j.molmet.2026.102459)
Supplement: Supplementary file 6 [file mmc4.pdf]

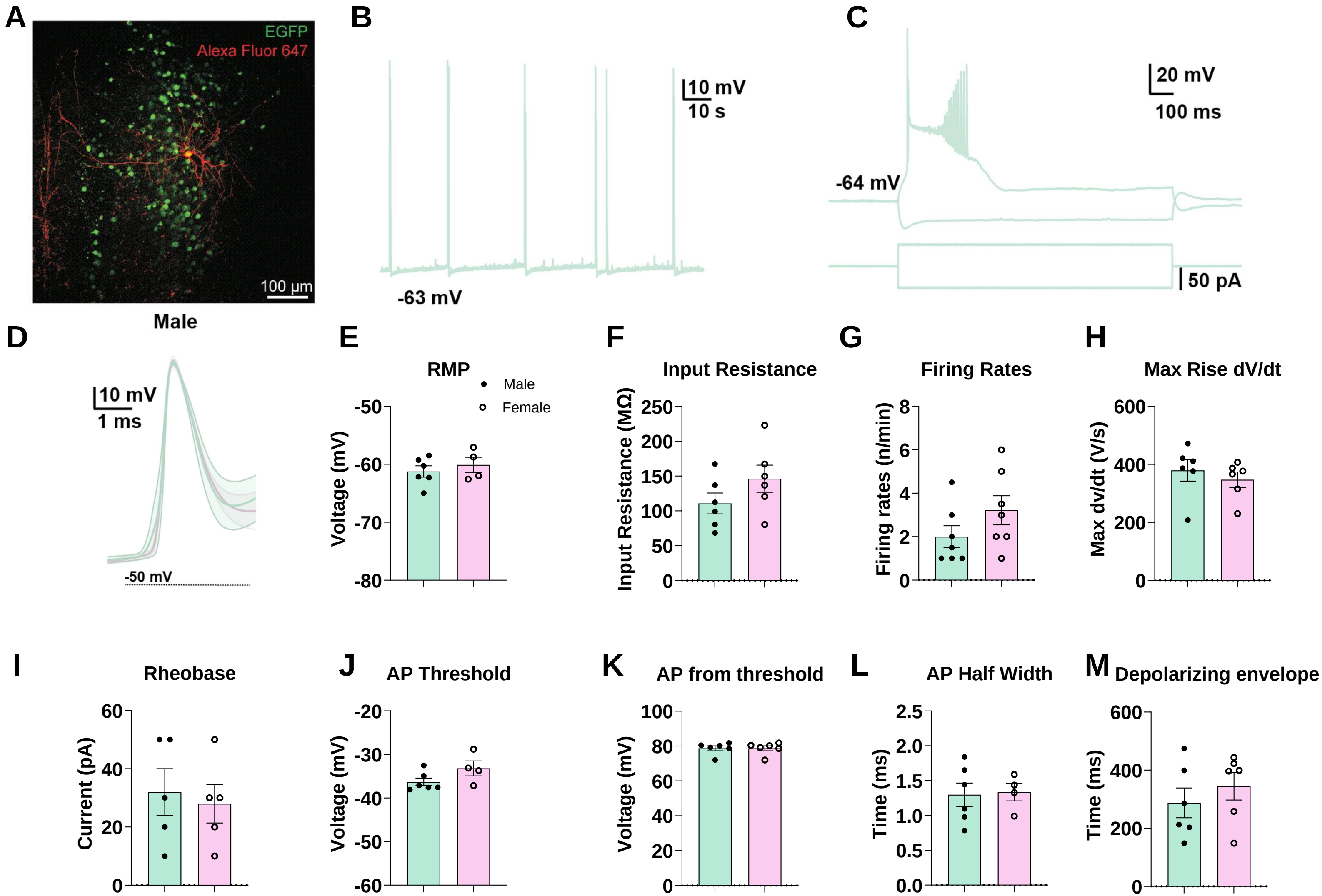
A
EGFP
Alexa Fluor 647
100 µm
Male
B
10 mV
10 s
-63 mV
C
20 mV
100 ms
-64 mV
50 pA
D
10 mV
1 ms
-50 mV
E
RMP
Male
Female
Voltage (mV)
F
Input Resistance
Input Resistance (MΩ)
G
Firing Rates
Firing rates (n/min)
H
Max Rise dV/dt
Max dv/dt (V/s)
I
Rheobase
Current (pA)
J
AP Threshold
Voltage (mV)
K
AP from threshold
Voltage (mV)
L
AP Half Width
Time (ms)
M
Depolarizing envelope
Time (ms)
