# Supplementary material for: A cortico-hypothalamic circuit regulates metabolism and stress resilience in a sex-specific manner
Source: Mol Metab. 2026 Oct 22;114:102459. doi: 10.1016/j.molmet.2026.102459 (PMC13755591; doi:10.1016/j.molmet.2026.102459)
Supplement: Supplementary file 7 [file mmc5.pdf]

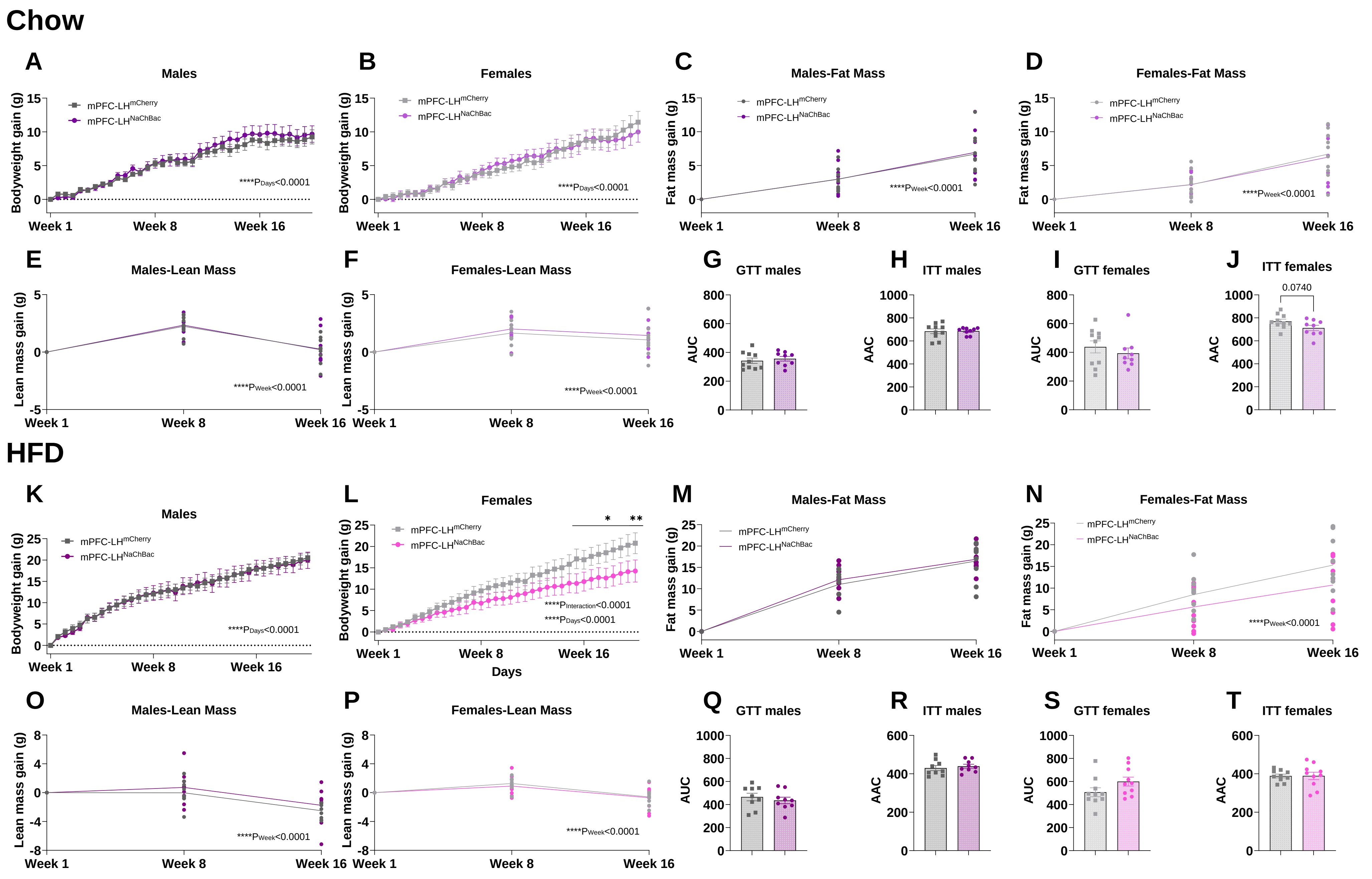

Chow
A
Males
Bodyweight gain (g)
mPFC-LH mCherry
mPFC-LH NaChBac
****P Days<0.0001
Week 1
Week 8
Week 16
B
Females
Bodyweight gain (g)
mPFC-LH mCherry
mPFC-LH NaChBac
****P Days<0.0001
C
Males-Fat Mass
Fat mass gain (g)
****P Week<0.0001
D
Females-Fat Mass
Fat mass gain (g)
****P Week<0.0001
E
Males-Lean Mass
Lean mass gain (g)
****P Week<0.0001
F
Females-Lean Mass
Lean mass gain (g)
****P Week<0.0001
G
GTT males
AUC
H
ITT males
AAC
I
GTT females
AUC
J
ITT females
AAC
0.0740
HFD
K
Males
Bodyweight gain (g)
mPFC-LH mCherry
mPFC-LH NaChBac
****P Days<0.0001
L
Females
Bodyweight gain (g)
mPFC-LH mCherry
mPFC-LH NaChBac
****P Interaction<0.0001
****P Days<0.0001
Days
M
Males-Fat Mass
Fat mass gain (g)
N
Females-Fat Mass
Fat mass gain (g)
****P Week<0.0001
O
Males-Lean Mass
Lean mass gain (g)
****P Week<0.0001
P
Females-Lean Mass
Lean mass gain (g)
****P Week<0.0001
Q
GTT males
AUC
R
ITT males
AAC
S
GTT females
AUC
T
ITT females
AAC
