# Supplementary material for: A cortico-hypothalamic circuit regulates metabolism and stress resilience in a sex-specific manner
Source: Mol Metab. 2026 Oct 22;114:102459. doi: 10.1016/j.molmet.2026.102459 (PMC13755591; doi:10.1016/j.molmet.2026.102459)
Supplement: Supplementary file 8 [file mmc6.pdf]

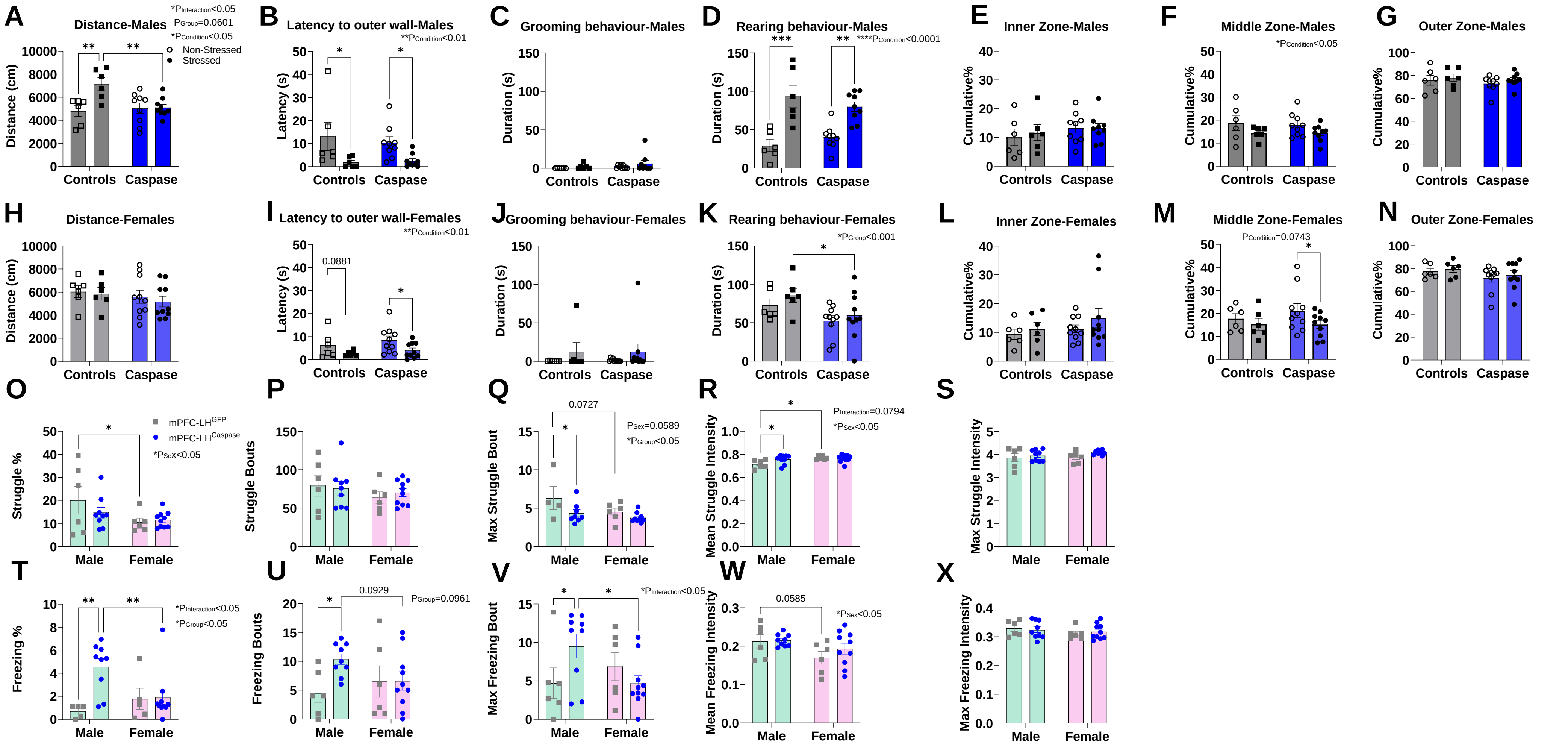

A
Distance-Males
*PInteraction<0.05
PGroup=0.0601
*PCondition<0.05
Non-Stressed
Stressed
Distance (cm)
Controls
Caspase
B
Latency to outer wall-Males
**PCondition<0.01
Latency (s)
C
Grooming behaviour-Males
Duration (s)
D
Rearing behaviour-Males
****PCondition<0.0001
E
Inner Zone-Males
Cumulative%
F
Middle Zone-Males
*PCondition<0.05
G
Outer Zone-Males
H
Distance-Females
I
Latency to outer wall-Females
**PCondition<0.01
0.0881
J
Grooming behaviour-Females
K
Rearing behaviour-Females
*PGroup<0.001
L
Inner Zone-Females
M
Middle Zone-Females
PCondition=0.0743
N
Outer Zone-Females
O
mPFC-LHGFP
mPFC-LHCaspase
*PSex<0.05
Struggle %
Male
Female
P
Struggle Bouts
Q
0.0727
PSex=0.0589
*PGroup<0.05
Max Struggle Bout
R
PInteraction=0.0794
*PSex<0.05
Mean Struggle Intensity
S
Max Struggle Intensity
T
*PInteraction<0.05
*PGroup<0.05
Freezing %
U
0.0929
PGroup=0.0961
Freezing Bouts
V
*PInteraction<0.05
Max Freezing Bout
W
0.0585
*PSex<0.05
Mean Freezing Intensity
X
Max Freezing Intensity
