# Supplementary material for: A cortico-hypothalamic circuit regulates metabolism and stress resilience in a sex-specific manner
Source: Mol Metab. 2026 Oct 22;114:102459. doi: 10.1016/j.molmet.2026.102459 (PMC13755591; doi:10.1016/j.molmet.2026.102459)
Supplement: Supplementary file 9 [file mmc7.pdf]

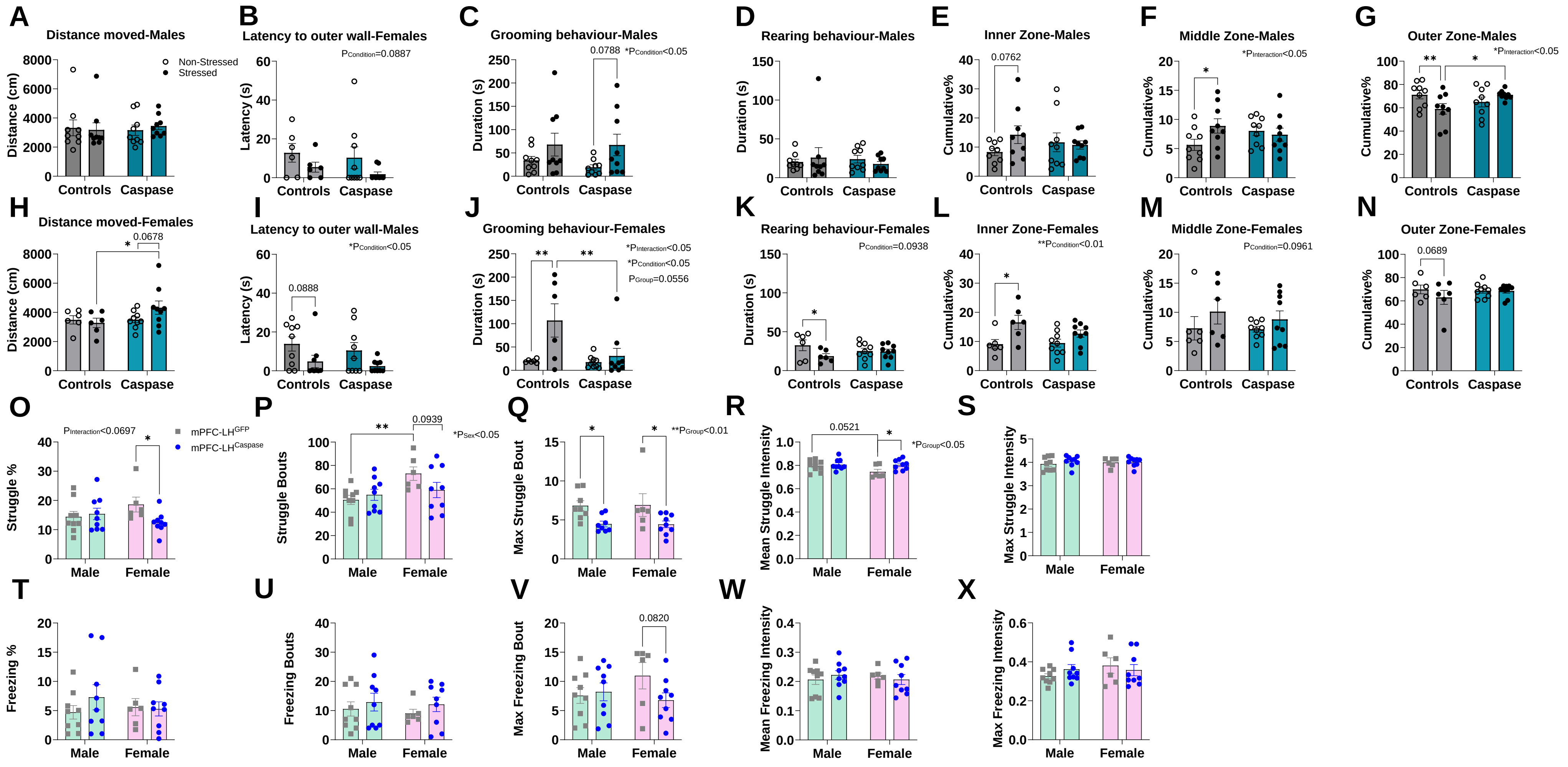

A
Distance moved-Males
Non-Stressed
Stressed
Distance (cm)
Controls
Caspase
B
Latency to outer wall-Females
PCondition=0.0887
Latency (s)
C
Grooming behaviour-Males
0.0788
*PCondition<0.05
Duration (s)
D
Rearing behaviour-Males
E
Inner Zone-Males
0.0762
Cumulative%
F
Middle Zone-Males
*PInteraction<0.05
G
Outer Zone-Males
*PInteraction<0.05
H
Distance moved-Females
0.0678
I
Latency to outer wall-Males
*PCondition<0.05
0.0888
J
Grooming behaviour-Females
*PInteraction<0.05
*PCondition<0.05
PGroup=0.0556
K
Rearing behaviour-Females
PCondition=0.0938
L
Inner Zone-Females
**PCondition<0.01
M
Middle Zone-Females
PCondition=0.0961
N
Outer Zone-Females
0.0689
O
PInteraction<0.0697
mPFC-LHGFP
mPFC-LHCaspase
Struggle %
Male
Female
P
0.0939
*PSex<0.05
Struggle Bouts
Q
**PGroup<0.01
Max Struggle Bout
R
0.0521
*PGroup<0.05
Mean Struggle Intensity
S
Max Struggle Intensity
T
Freezing %
U
Freezing Bouts
V
0.0820
Max Freezing Bout
W
Mean Freezing Intensity
X
Max Freezing Intensity
