# Supplementary material for: A cortico-hypothalamic circuit regulates metabolism and stress resilience in a sex-specific manner
Source: Mol Metab. 2026 Oct 22;114:102459. doi: 10.1016/j.molmet.2026.102459 (PMC13755591; doi:10.1016/j.molmet.2026.102459)
Supplement: Supplementary file 10 [file mmc8.pdf]

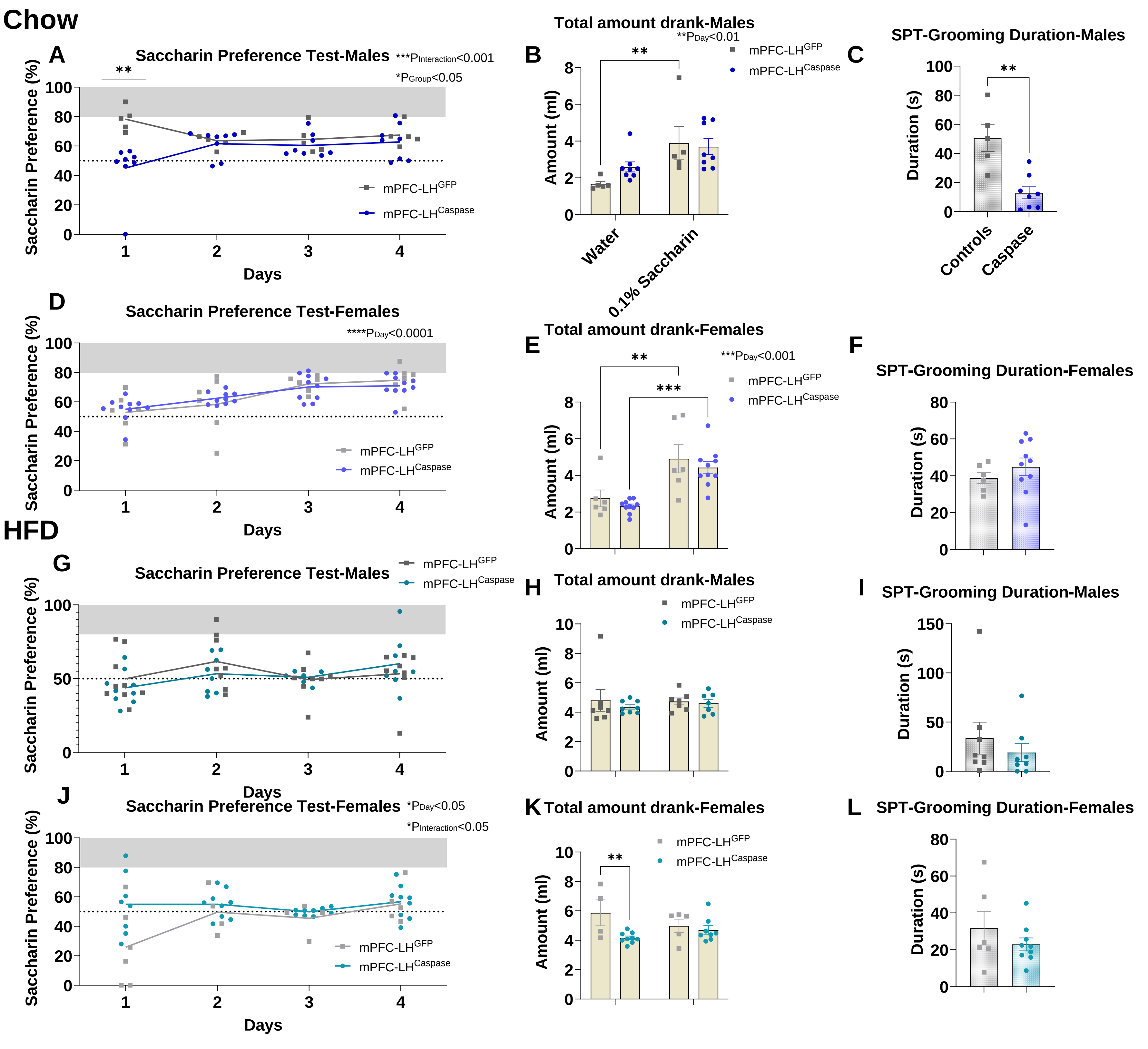

Chow
A
Saccharin Preference Test-Males
***P Interaction<0.001
*P Group<0.05
Saccharin Preference (%)
Days
mPFC-LH GFP
mPFC-LH Caspase
B
Total amount drank-Males
**P Day<0.01
Amount (ml)
Water
0.1% Saccharin
C
SPT-Grooming Duration-Males
Duration (s)
Controls
Caspase
D
Saccharin Preference Test-Females
****P Day<0.0001
E
Total amount drank-Females
***P Day<0.001
F
SPT-Grooming Duration-Females
HFD
G
Saccharin Preference Test-Males
H
Total amount drank-Males
I
SPT-Grooming Duration-Males
J
Saccharin Preference Test-Females
*P Day<0.05
*P Interaction<0.05
K
Total amount drank-Females
L
SPT-Grooming Duration-Females
