# Supplementary material for: A cortico-hypothalamic circuit regulates metabolism and stress resilience in a sex-specific manner
Source: Mol Metab. 2026 Oct 22;114:102459. doi: 10.1016/j.molmet.2026.102459 (PMC13755591; doi:10.1016/j.molmet.2026.102459)
Supplement: Supplementary file 11 [file mmc9.pdf]

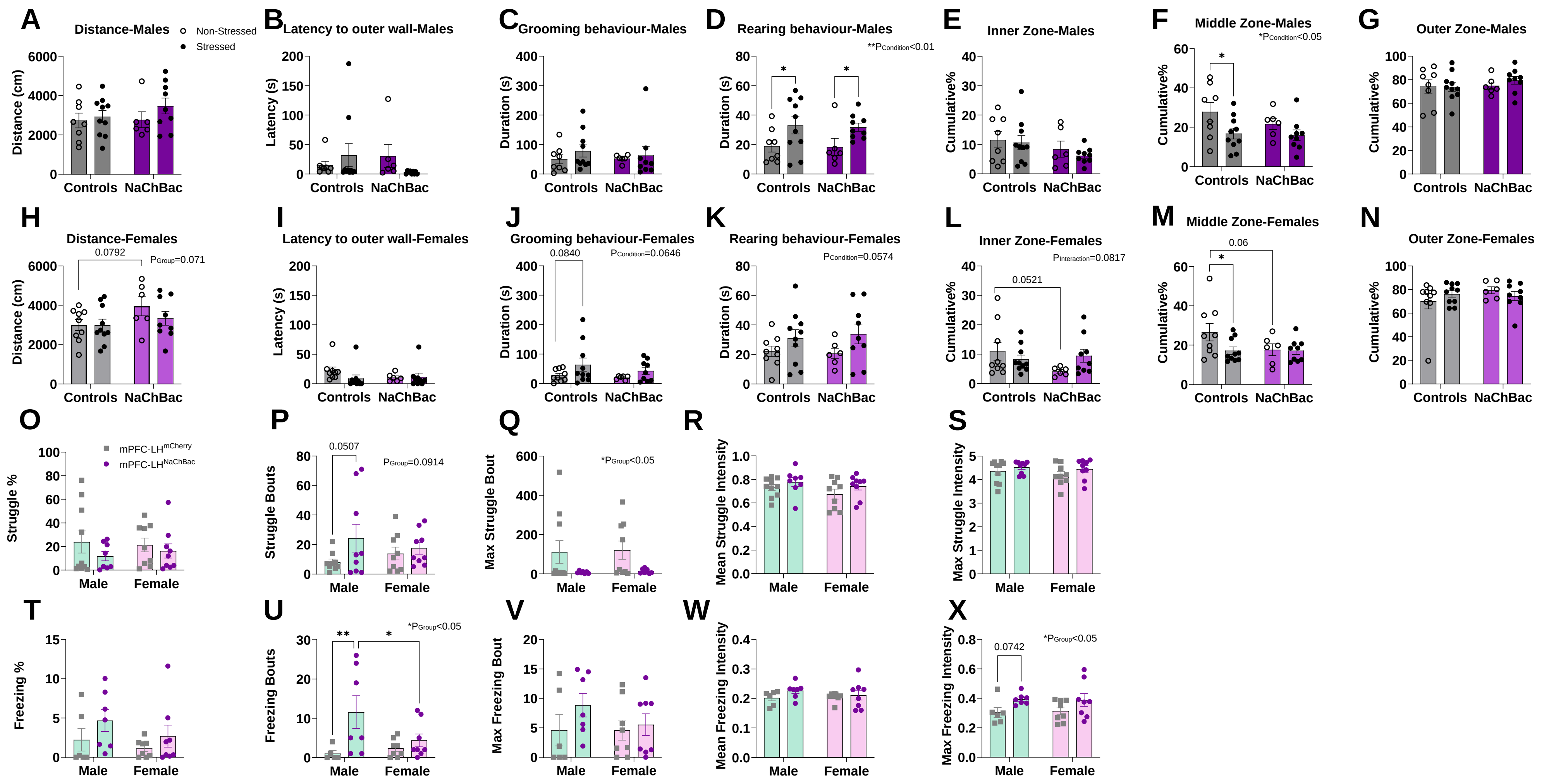
A
Distance-Males
Non-Stressed
Stressed
Distance (cm)
Controls
NaChBac
B
Latency to outer wall-Males
Latency (s)
C
Grooming behaviour-Males
Duration (s)
D
Rearing behaviour-Males
**PCondition<0.01
E
Inner Zone-Males
Cumulative%
F
Middle Zone-Males
*PCondition<0.05
G
Outer Zone-Males
H
Distance-Females
0.0792
PGroup=0.071
I
Latency to outer wall-Females
J
Grooming behaviour-Females
0.0840
PCondition=0.0646
K
Rearing behaviour-Females
PCondition=0.0574
L
Inner Zone-Females
PInteraction=0.0817
0.0521
M
Middle Zone-Females
0.06
N
Outer Zone-Females
O
mPFC-LHmCherry
mPFC-LHNaChBac
Struggle %
Male
Female
P
Struggle Bouts
0.0507
PGroup=0.0914
Q
Max Struggle Bout
*PGroup<0.05
R
Mean Struggle Intensity
S
Max Struggle Intensity
T
Freezing %
U
Freezing Bouts
*PGroup<0.05
V
Max Freezing Bout
W
Mean Freezing Intensity
X
Max Freezing Intensity
0.0742
*PGroup<0.05
